# Mechanical compaction alters microstructural and magnetic resonance imaging properties of acute ischemic stroke clots

**DOI:** 10.1177/0271678X261465841

**Published:** 2026-06-25

**Authors:** Cody J Kubicki, Thomas Neuberger, Scott D Simon, Keefe B Manning

**Affiliations:** 1 Department of Biomedical Engineering, The Pennsylvania State University, University Park, PA, USA; 2 Huck Institutes of the Life Sciences, The Pennsylvania State University, University Park, PA, USA; 3 Department of Neurosurgery, Penn State College of Medicine, Hershey, PA, USA; 4 Department of Surgery, Penn State College of Medicine, Hershey, PA, USA

**Keywords:** Acute ischemic stroke, thrombolytic therapy, thromboembolism, magnetic resonance imaging, susceptibility vessel sign

## Abstract

Thrombolytic recanalization rates in acute ischemic stroke (AIS) patients remain low. Magnetic resonance susceptibility vessel sign has been correlated with reduced thrombolytic susceptibility. However, the driving mechanisms have not been clarified. In the present study, high-field magnetic resonance imaging is used to quantify relaxation rates and compaction of embolus analogs undergoing simulated AIS with varying pressures. Microscopy and perfusion tests are used to study the effect of compaction on clot microstructure and permeability, respectively. The results show that pressure dependent compaction alters clot microstructural characteristics and increases the R2 relaxation rate. Following compaction, the R2 in red blood cell (RBC) rich clots increases up to 224%, corresponding to a twofold increase in RBC volume fraction and polyhedrocyte formation. RBC and fibrin dominant clot permeabilities decrease by 78% and 97%, respectively, when compaction pressure increases 50 mmHg. Compacted RBC rich clots are the most likely to present with susceptibility vessel sign and have the lowest permeability. Our results suggest that increased clot constituent density due to compaction can lead to susceptibility vessel sign and elevated thrombolytic resistance by limiting thrombolytic perfusion. This is the first study to evaluate the clinical relevance of clot compaction during AIS using multiple imaging techniques.

## Graphical abstract

**Graphical Abstract:**
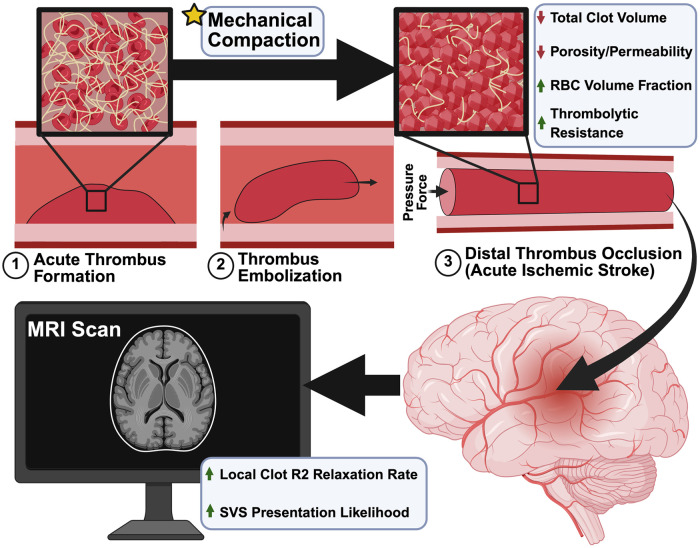

## Introduction

Acute ischemic stroke (AIS) is a leading cause of disability and mortality globally.^1^ AIS is often attributed to thromboembolic events that initiate when clots form in the upstream circulation due to an existing vascular disease, cardiac dysfunction, or implanted device, and then detach and embolize to the smaller cerebral vessels where they become lodged causing complete or partial vessel occlusion. Intravenous thrombolytic therapy (IVT) is a common AIS treatment methods that involves systemic infusion of recombinant tissue plasminogen activator (r-tPA; i.e. Alteplase/Activase) or a modified r-tPA thrombolytic agent, such as Tenecteplase, to dissolve the occlusive clot biochemically. Despite the prevalence of IVT for AIS patient treatment, complete recanalization rates remain low for certain cases, which results in poor short and long-term functional outcomes.^2–4^ High incidence of suboptimal outcomes may be explained by the physical properties of the clot, which are often occult on computed tomography (CT) imaging, the most common first line imaging modality for AIS. To address this deficiency, recent research has focused on developing new imaging techniques for AIS clot characterization and IVT efficacy determination.^5–10^ Certain clot properties, including histological composition and porosity, can significantly influence IVT susceptibility.^11–15^ Therefore, methods leveraging data from current imaging modalities to identify these critical clot properties in vivo would permit more informed treatment determination based on IVT success potential with minimal delay to the time-sensitive clinical workflow. This type of image-based treatment determination approach could improve vessel recanalization times and success rates that are imperative to favorable outcomes.

Magnetic resonance imaging (MRI) and CT scans are both common for AIS evaluation. Although CT is the most prevalent diagnostic imaging method for AIS, MRI has emerged in some neurological centers as the first line imaging tool for suspected AIS due its distinct advantages in clot identification through T2* (T2*-w) or susceptibility weighted imaging (SWI) and brain damage assessment through perfusion (PWI) and diffusion weighted imaging (DWI).^16,17^ Susceptibility vessel sign (SVS) presenting in an MRI scan is a common indicator for AIS. SVS is characterized by a hypointense region at the occlusive clot location that appears in SWI and is hypothesized to arise because of the direct correlation between the transverse R2* relaxation rate (rate of magnetic spin dephasing) and clot constituent density, particularly red blood cells (RBCs).^18–21^ SVS is associated with large vessel occlusion (LVO), poor patient outcomes, lower vessel perfusion scores, and increased thrombolytic resistance.^22,23^ Although statistical correlations between SVS and clinical therapeutic outcomes have been identified, the mechanisms behind these relationships and the conditions that lead to the presence of SVS in certain patients remain unclear. Leveraging the versatility of MRI scans to expand the scope of the initial clinical evaluation beyond a binary SVS positive or negative diagnosis to provide a more detailed clot characterization could improve our understanding of the link between MR signal and clinical outcomes.

Current research has largely ignored the influence of the mechanical lodging event during AIS. We hypothesize that (1) there is clot compaction that occurs because of the initial cerebral vessel occlusion and (2) compaction contributes to higher clot constituent density, which increases SVS likelihood and thrombolytic resistance. To test this hypothesis, we replicated AIS in vitro within a high-field MRI scanner to quantify MR properties of two embolus analog (EA) types under different compaction pressures. We then related the MR property changes to microstructural changes that directly influence thrombolytic susceptibility by performing histological imaging, scanning electron microscopy (SEM) imaging, and permeability quantification. Comparisons between the microstructures of the EAs and clots retrieved from AIS patients by endovascular thrombectomy (EVT) were completed to demonstrate the clinical relevance of our findings.

## Material and methods

### Human blood collection and embolus analog formation

**Figure 1.**
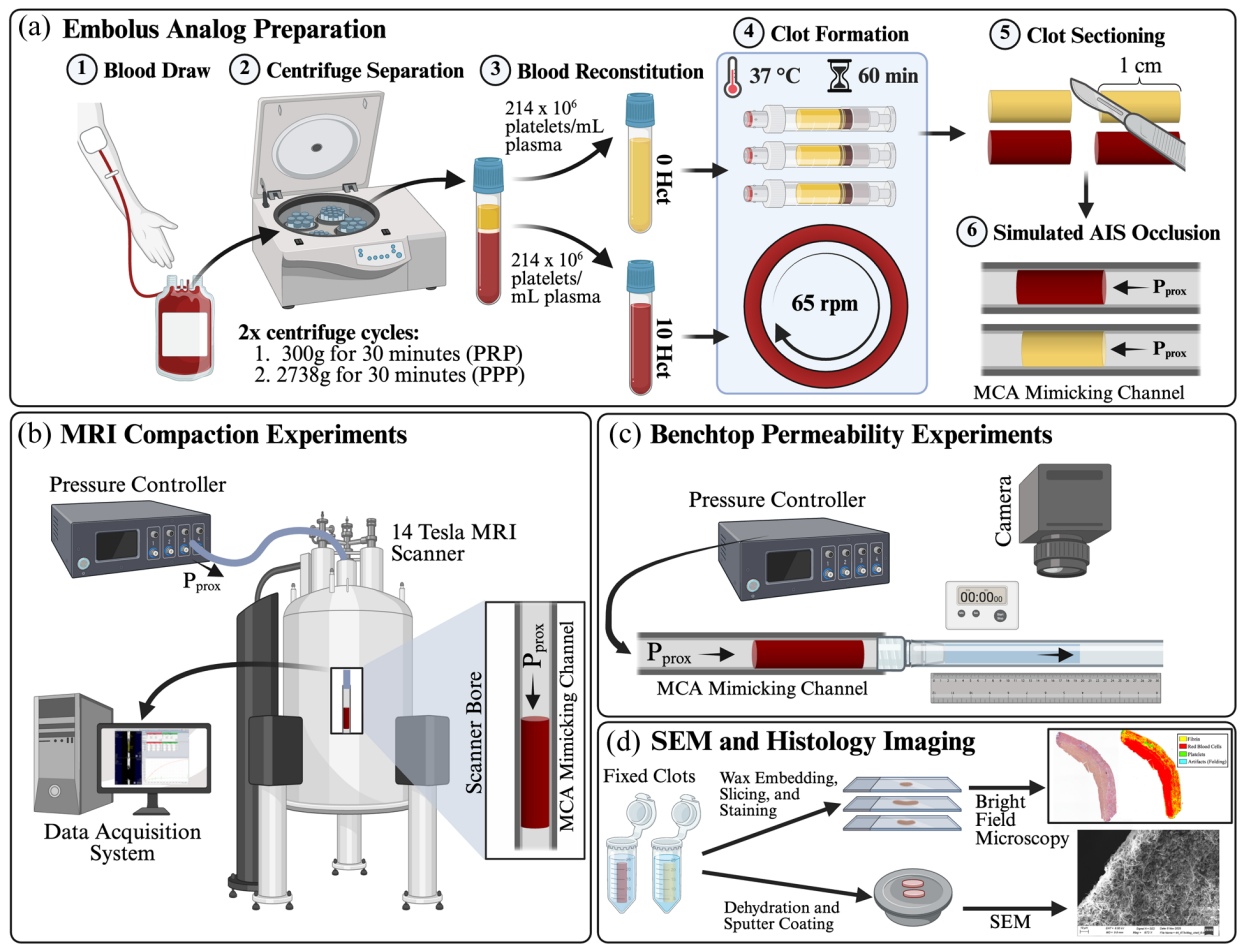
Illustrations detailing the methods used for (a) generating the EAs used in each of the experiments: 1—blood draw from healthy volunteers, 2—two-step blood separation by centrifugation, 3—reconstitution of separated blood into 0 and 10 Hct samples with controlled platelet concentration, 4—formation of 10 Hct clots in a Chandler Loop and 0 Hct clots in static syringes, 5—slicing formed clots into 1 cm long EAs, and 6—injecting the EAs into MCA mimicking channels with controlled pressure to simulate AIS, (b) scanning the EAs under varying pressure using MRI, (c) measuring the EA permeability using a benchtop permeation tracking system, and (d) imaging the microstructure of the EAs using SEM and histology.

Anticoagulated (0.32% sodium citrate) blood was collected from healthy human volunteers following a protocol in accordance with ethical guidelines outlined by the Belmont Report and Declaration of Helsinki and approved by the Penn State Internal Review Board (IRB). Informed consent was obtained from all participants. The blood was separated into platelet rich plasma (PRP) and platelet poor plasma (PPP) by two centrifugation cycles. Reconstituted 10 and 0 Hct blood samples were made with a controlled platelet count (214 × 10^6^ platelets/mL). EAs were formed from the reconstituted samples by adding CaCl_2_ (20 mM) and human thrombin (BioPharm Laboratories, Bluffdale, UT, USA; 0.25 U/mL). The 10 Hct EAs were formed in a Chandler Loop and the 0 Hct EAs were formed in 3 mL syringes for 1 h at 37 °C. After formation, the EAs were sliced into 1 cm long samples and placed into phosphate buffered saline (PBS) in preparation for the simulated AIS experiments (Figure 1(a)). Two samples of each EA type were placed into 4% paraformaldehyde (PFA) as an uncompacted sample for histology and SEM. All EAs were formed within 6 h of blood donation.

### Hematocrit and plasma calibration curve generation using MRI

Separated RBCs were used to form 40%, 60%, and 80% RBC solutions in PBS. Similarly, 20%, 40%, 60%, and 80% plasma solutions were formed from the separated PRP. These solutions were scanned in a 14 Tesla vertical wide bore microimaging MRI system (Avance NEO; Bruker, Billerica, MA, USA) using a quadrature driven bird cage radiofrequency (RF) resonator. Images were acquired using a rapid acquisition with relaxation enhancement and variable repetition time (RARE–VTR) spin echo pulse sequence and processed using an in-house MATLAB (MathWorks, Inc., Natick, MA, USA) script (details in Supplemental Methods SM1) to quantify the relaxation rates and generate a calibration curve.

### MRI scan acquisition and processing during simulated AIS

RARE–VTR and three-dimensional fast low-angle shot (3D-FLASH) scans (pulse sequence parameters in Supplemental Table ST1) of an EA in PBS were acquired to quantify the MR relaxation rates and volume of the uncompacted EA, respectively. Following the uncompacted scans, the EA was placed into the proximal end of a 3D printed middle cerebral artery (MCA) mimicking channel with PBS and positioned at the magnetic isocenter of the scanner. The channel was designed with a lumen that matches the taper angle (0.9°) and internal diameters of the proximal M1-MCA segment (3.1 mm) to the distal M2-MCA segment (1.5 mm).^24,25^ The MCA was chosen as the target vessel for simulating AIS because it is the most common location for clinical LVO cases.^3,26,27^ Tubing connected the proximal end of the MCA mimicking channel to a pneumatic pressure controller (OB1-MK4; Elveflow, Paris, France) that enabled precise proximal pressure control (Figure 1(b)). The system was pressurized by filtered atmospheric air, and the experiments were performed at room temperature (23 °C) with an average humidity of 36%. Each clot was imaged sequentially under 25, 50, 75, 100, and 125 mmHg of pressure, which span the physiological range of pressure drops experienced by occlusive AIS clots in vivo.^28,29^ A localizer scan was acquired between each pressure increase to locate and center the EA position as it was pushed distally in the channel due to the pressure changes. After positioning, a 5-min delay period was allowed to pass prior to acquisition of the 3D-FLASH and RARE–VTR scans. In total, all scans for the range of compaction pressures applied to each clot took approximately 40 min to acquire. Following MRI scan completion, the compacted EA was removed from the channel and fixed in 4% PFA for histology and SEM imaging. MRI experiments were performed on one 0 and one 10 Hct EA for each donor and were repeated for six different donors (*n* = 6 total for each EA type; donor demographic information in Supplemental Table ST2).

The 3D-FLASH sequence images were manually segmented and interpolated in Avizo (Thermo Fisher Scientific, Waltham, MA, USA) to generate volumetric reconstructions for volume calculation and morphological assessment. The RARE–VTR images were used to calculate the R1 and R2 averages, spatial maps, and histograms in the EA tissue (details in Supplemental Methods SM1). The R2 values were input into the calibration equations to estimate the plasma protein or RBC volume fraction.

### Clot permeability quantification

Formed EAs were injected with PBS into MCA mimicking channels under either 25 or 75 mmHg of proximal pressure, representing the approximate minimum (two standard deviations below average) and average pressure drop across an AIS clot in vivo, respectively.^28^ A high-resolution recording was acquired to track fluid movement while constant pressure was applied (Figure 1(c)). Using still frames from the recording and ImageJ (NIH, Bethesda, MD, USA), the permeability was quantified using Darcy’s Law by measuring the mean flow rate, clot length, and clot diameter. A Vilastic-3 viscoelasticity analyzer (Vilastic Scientific, Inc., Austin, TX, USA) was used to measure the permeating fluid viscosity. Following the permeability experiments, EAs were removed from the channels and fixed in 4% PFA for histology and SEM imaging. Three EAs of each type were tested for both pressure conditions using six different donors (*n* = 18 for each EA type and pressure; donor demographic details in Supplemental Table ST3).

### Scanning electron microscopy

Fixed EAs were washed with PBS and dehydrated by sequential submersion in increasing concentrations of ethanol and hexamethyldisilazane (HMDS). The dehydrated clots were sliced to expose the inner core and adhered to specimen stubs with the core oriented away from the adhesion surface. The adhered samples were sputter coated with 6 nm of iridium (Leica EM ACE600; Deerfield, IL, USA) and imaged using a field emission scanning electron microscope (Zeiss SIGMA VP-FESEM; White Plains, NY, USA).

### Histological staining and imaging

Fixed EAs were dehydrated in increasing concentrations of ethanol, embedded in paraffin wax, and sliced into 6 µm thick sections using a microtome (Shandon Finesse ME; Thermo Fisher Scientific, Waltham, MA, USA). The sliced samples were stained using a Carstairs staining protocol to distinguish the primary clot constituents, including RBCs, fibrin, platelets, and collagen (Figure 1(d)).^30^ Stained samples were imaged under bright field using an Olympus BX61 microscope (Evident Scientific, Waltham, MA, USA) using 4× and 20× objective lenses. The high magnification images were processed in Orbit Image Analysis to quantify clot constituent fractions and porosity (details in Supplemental Methods SM2).^31^

### Stroke patient clot sample extraction and preparation

Following an approved Penn State IRB protocol, clots extracted from AIS patients at Hershey Medical Center by EVT were rinsed in saline and fixed in 4% PFA at 4 °C. Patient demographic data were recorded for each retrieved clot (donor demographic details in Supplemental Table ST4). For all cases, the systolic blood pressure was maintained between 140 and 180 mmHg during the EVT procedure. Fixed patient samples were cut into multiple parts that were randomly assigned to histological or SEM imaging so that each clot was imaged with both microscopy techniques. Patient clots were prepped and imaged using the same protocols described previously. A total of 12 extracted patient clots were imaged.

### Statistical analysis

All data sample distributions were checked for normality using a Shapiro–Wilk test. Normal sample distributions are reported as the sample mean ± standard deviation, unless otherwise specified. Non-normal sample distributions are reported as the sample median and interquartile range (IQR). A paired *t*-test was used to compare MR relaxation rates and EA volumes before and after compaction. A non-parametric Kruskal–Wallis test with a Tukey post-hoc analysis was used to compare histological constituents and permeabilities across clot types and compaction pressures. Simple univariate linear regression models were used to determine the relationship between MR relaxation rates, clot volume, and compaction pressure. The regression model results are presented as the estimated coefficient ± the standard error of the coefficient and the determination coefficient (*R*^2^). All statistical analyses were performed using MATLAB (v2024b). A *p* < 0.05 was deemed statistically significant.

## Results

### R1 and R2 relaxation rate quantification and constituent estimates

Both calibration curves showed a strong (*R*^2^ *>* 0.99) positive linear correlation between the average R2 relaxation rate and the blood component volume fraction (Supplemental Methods SM1), which is consistent with prior studies.^32–34^ The R2 values of the RBC samples were higher consistently than those of the plasma samples. R2 was more sensitive to changes in RBC volume fraction, as indicated by the calibration curve slopes of 0.916 ± 0.117 s^−1^/% RBC (*R*^2^ = 0.79) and 0.091 ± 0.004 s^−1^/% plasma (*R*^2^ = 0.98). The RBC samples had greater intra-donor variability compared to the plasma samples, particularly at higher hematocrits.

**Figure 2.**
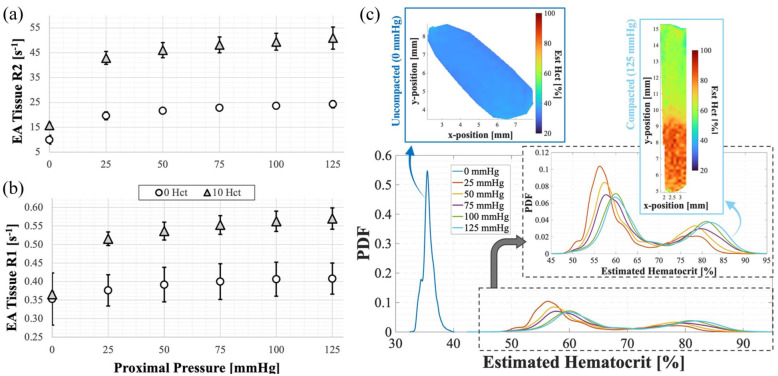
(a) Average transverse R2 relaxation rate and (b) average longitudinal R1 relaxation rate within the clot tissue measured while under different proximal compaction pressures in the MCA mimicking channel and measured by the 14 Tesla MRI scanner. Note that the *y*-axis scale is different between (a) and (b) due to the difference in magnitude between the two relaxation rates. Error bars represent the standard deviation (*n* = 6 for each EA type and pressure) and (c) PDF distribution curves of the pixel-wise estimated hematocrit within the clot tissue ROI for a representative 10 Hct sample under all six compaction pressure conditions. The inset PDF plot shows the same plot focused on only the compaction cases to highlight the smaller differences between the compaction pressure conditions. The overlaid contour plots show the estimated hematocrit spatial distributions of the two respective conditions indicated (0 and 125 mmHg). PDF: probability density function.

The average R2 of both EA types increased significantly following compaction (Figure 2(a)). The 10 Hct EA R2 increased from 15.7 ± 1.0 s^−1^ at 0 mmHg to 42.9 ± 2.6 s^−1^ at 25 mmHg (*p* < 1E-6), which corresponded to an 82% increase in average RBC volume fraction from 37% to 67% calculated from the calibration curve. The R2 increased further to 50.9 ± 4.5 s^−1^ as the compaction pressure increased to 125 mmHg, a 224% increase from the uncompacted state. The 0 Hct EA R2 increased from 10.0 ± 1.8 s^−1^ at 0 mmHg to 19.7 ± 1.6 s^−1^ at 25 mmHg (*p* < 1E-5), corresponding to a 132% increase in estimated constituent fraction, primarily fibrin, from 74% and 172%. At the maximum 125 mmHg compaction pressure, the 0 Hct EA average R2 increased to 24.3 ± 1.4 s^−1^. The R1 value in the 10 Hct clots also increased significantly after initial compaction from 0 to 25 mmHg (0.37 ± 0.01 vs 0.52 ± 0.02 s^−1^, *p* *<* 1E-5), which was not observed in the 0 Hct clots (0.35 ± 0.07 vs 0.38 ± 0.04 s^−1^, *p* = 0.103; Figure 2(b)). The plasma constituent fraction estimates were higher and exceeded 100% because the calibration samples did not account for platelet mediated clot contraction and fibrin polymerization, which further elevate R2 beyond that of plasma alone.^35^ Therefore, these constituent volume fraction estimates provide better insights into relative shifts between compaction states, as opposed to true magnitudes of the solid volume fraction.

Higher compaction pressures resulted in a significant increase in R2 for both EA types. The regression analysis estimated an R2 increase of 0.08 ± 0.02 s^−1^/mmHg (*R*^2^ = 0.43, *p* < 1E-4) in the 10 Hct EAs and 0.05 ± 0.01 s^−1^/mmHg (*R*^2^ = 0.60, *p* < 1E-6) in the 0 Hct EAs. These correlations corresponded to a 1% increase in RBC fraction for every 11.8 mmHg of added pressure to the 10 Hct EAs and a 1% increase in plasma constituent fraction for every 2.2 mmHg of added pressure to the 0 Hct EAs. The regression model for the R1 relaxation rates showed a significant relationship between pressure and R1 for the 10 Hct EAs, 5.4E-4 ± 1.2E-4 s^−1^/mmHg (*R*^2^ = 0.385, *p* < 0.001). However, this relationship was not significant for the 0 Hct EAs, 3.1E-4 ± 2.2E-4 s^−1^/mmHg (*R*^2^ = 0.067, *p* = 0.168).

The probability density function histograms of the estimated RBC volume fraction showed a sharp increase in hematocrit throughout the clot sample following compaction (Figure 2(c)). The histogram distributions continued to shift right as the pressure increased, indicating higher average RBC fraction as the clot compacted further. The uncompacted clot hematocrit values were homogeneous and normally distributed, whereas the compacted sample histograms were skewed typically to the right or had bimodal distributions. In some cases, particularly at higher compaction pressures, the tails of the estimated hematocrit distributions reached values above 100% (Supplemental Figure SF1). The highest estimated hematocrit values were present in the distal end of the clot. There were regions in the proximal end of the clot where the local hematocrit was lower than surrounding regions, which appeared to result from RBC expulsion into the fluid column during compaction (Supplemental Figure SF2).

### Clot volume and morphology changes during compaction

**Figure 3.**
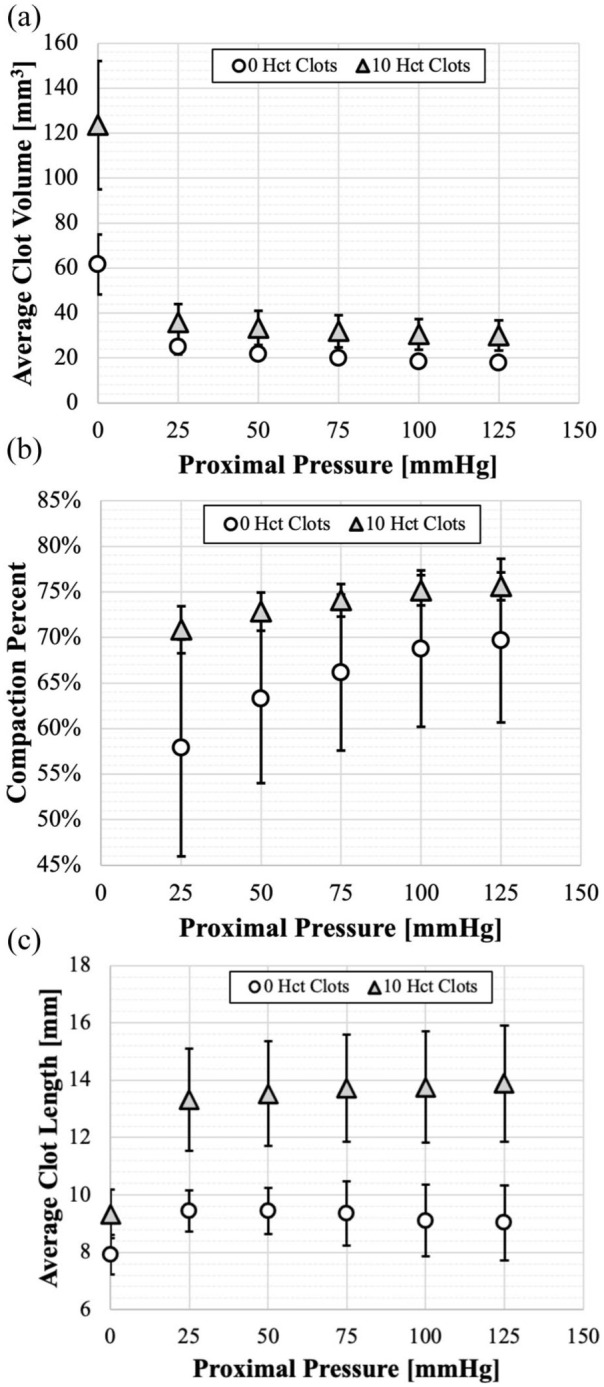
Scatter plots showing the relationship between the proximal compaction pressure and (a) the average clot volume, (b) the compaction percentage relative to the original uncompacted state, and (c) the total clot length. Error bars represent the standard deviation (*n* = 6 for each EA type and pressure).

The EA volume was reduced, and clot length was increased significantly following compaction for both EA types (Figure 3). The significant EA morphological changes were visualized in the 3D reconstructions as the clot was pushed distally in the tapered MCA mimicking channel (Supplemental Figure SF3). The 10 Hct EA volume and length went from 123.6 ± 28.5 mm^3^ and 9.3 ± 0.9 mm uncompacted to 35.8 ± 8.1 mm^3^ and 13.3 ± 1.8 mm when compacted under 25 mmHg pressure (*p* *<* 0.001), respectively. Similarly, the 0 Hct EA volume was reduced from 61.6 ± 13.4 to 24.9 ± 3.3 mm^3^ (*p* *<* 0.005), and length was increased from 7.9 ± 0.7 to 9.5 ± 0.7 mm (*p* *<* 0.01). The 0 Hct EAs were smaller consistently, likely due to the increased level of initial contraction and had a lower relative volume compaction percentage compared to the 10 Hct EAs. The relative compaction percentage of the 10 Hct EAs ranged from 70.9% ± 2.6% at 25 mmHg to 75.6% ± 1.5% at 125 mmHg. In comparison, the 0 Hct clots had relative compaction percentages of 57.9% ± 11.9% and 69.7% ± 9.0% at 25 and 125 mmHg, respectively (Figure 3(b)). There was a plateauing effect at 100 mmHg for both EA types, where the relative compaction increase was less significant as the pressure increased.

### Clot microstructural characterization under microscopy

**Figure 4.**
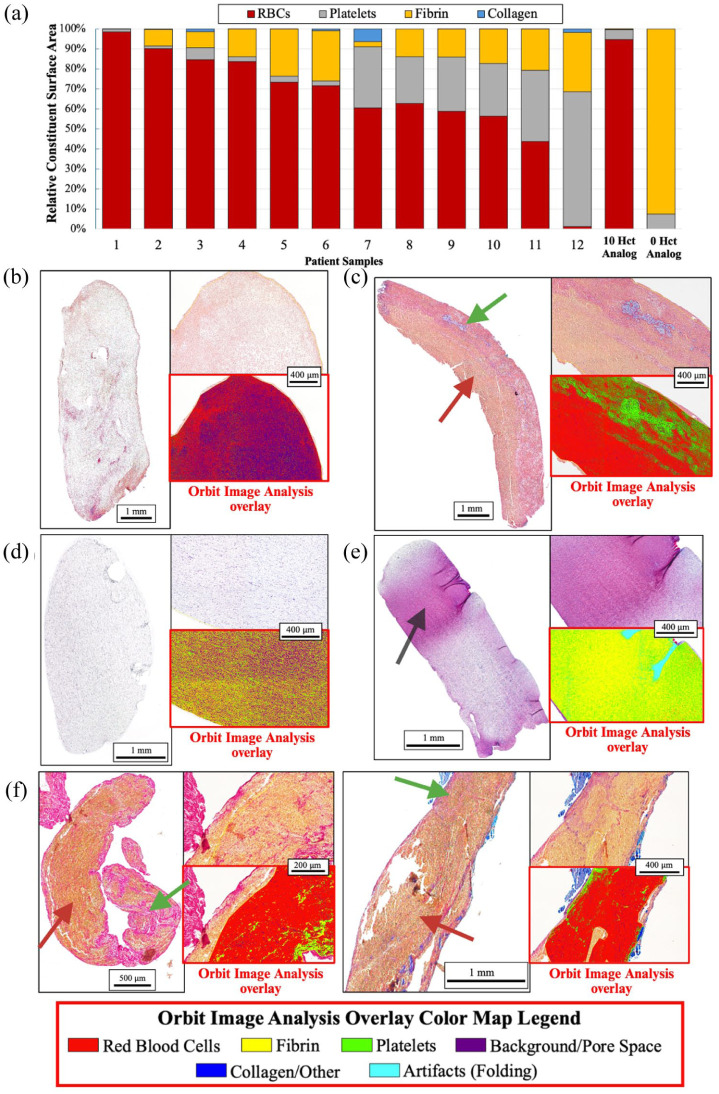
(a) A bar graph summarizing the relative constituent surface area fractions within each of the 12 patient clots extracted by EVT and the average constituent distributions in the in vitro EAs (*n* = 3 for each EA type), (b–g) histology images taken using brightfield microscopy after clots were stained using Carstairs method. The images were taken from representative examples of (b, c) 10 Hct and (d, e) 0 Hct in vitro EAs in both the (b, d) uncompacted and (c, e) 125 mmHg compacted state, as well as from (f) two RBC-rich extracted patient clots. RBCs appear yellow/red-orange, fibrin appears red/light purple, platelets appear a light blue-gray to dark purple, and collagen appears bright blue. The images on the left of each subfigure show the bulk clot structure imaged using a 4× objective lens. The top-right images show a smaller magnified section of the clot using a 20× objective lens that was used to segment, map, and quantify the local clot constituents and porosity using Orbit Image Analysis software. The output spatial maps of the constituent distribution are shown in the corresponding bottom-right images. The legend at the bottom indicates the individual components that each color represents in these processed spatial maps. The red and green arrows highlight RBC and fibrin/platelet dominant regions, respectively. The black arrow highlights a region of elevated compaction in the 0 Hct EA that arises due to folding at the distal end.

The constituent fractions within the 12 extracted patient clots varied widely and included RBC rich, fibrin/platelet rich, and mixed clot types (Figure 4(a)). The median RBC, fibrin, and platelet compositions across all the extracted patient samples were 74.8% (IQR: 56.4%–87.5%), 13.3% (IQR: 7.7%–20.8%), and 5.3% (IQR: 2.6%–28.8%), respectively. The in vitro EA compositions had less variability between samples due to the control over blood composition and environmental conditions during formation. The 0 Hct EAs were composed of 94.5% (IQR: 92.1%–97.9%) fibrin and 5.5% (IQR: 2.1%–7.9%) platelets with negligible RBC content. Whereas the 10 Hct EA composition was 95.4% (IQR: 90.7%–99.7%) RBCs, 0.1% (IQR: 0.0%–1.3%) fibrin, and 3.6% (IQR: 0.3%–9.3%) platelets.

The uncompacted 10 and 0 Hct EAs had average histology measured porosities of 63.0% ± 6.4% and 42.4% ± 16.5%, respectively, which were significantly higher than the 4.5% ± 4.4% porosity of the extracted patient clots. Compaction of the 10 and 0 Hct EAs at 125 mmHg significantly reduced the porosities to 17.5% ± 8.3% (*p* < 0.05) and 8.0% ± 10.3% (*p* < 0.01), respectively. Following compaction, there was no significant difference between the 0 Hct EA and patient clot porosities (*p* = 0.91). Although the porosity estimates for the 10 Hct clots were still significantly higher than the patient clots (*p* < 0.05), the compacted samples were more representative of the extracted patient clots than the uncompacted EAs (Supplemental Table ST5).

The porosity reduction effect due to compaction was observed qualitatively in the histology images (Figure 4). The overall appearances of the 125 mmHg compacted 10 Hct EAs (Figure 4(c)) were more like the two RBC-rich patient clots (Figure 4(f)). Both had high stained constituent densities and heterogeneous distribution patterns. Certain regions had a higher RBC concentration (Figure 4, red arrows) and others had higher fibrin/platelet content (Figure 4, green arrows). In contrast, the uncompacted 10 Hct EA (Figure 4(b)) had a lower stained constituent density with a more homogeneous distribution. Similar phenomena were observed in the 0 Hct EAs. The uncompacted 0 Hct EA (Figure 4(d)) had a low constituent density that resulted in a faint appearance of the stained components throughout, whereas the 125 mmHg compacted 0 Hct EA (Figure 4(e)) appeared darker, indicating a constituent density increase. Multiple 0 Hct EAs displayed a distinct darker band near the distal end of the clot (Figure 4(e), black arrow) that corresponded with a region where the clot folded in on itself, further compacting the clot and increasing constituent density. Additional high magnification images highlighting the individual constituents and microstructural compaction are included in Supplemental Figure SF4.

**Figure 5.**
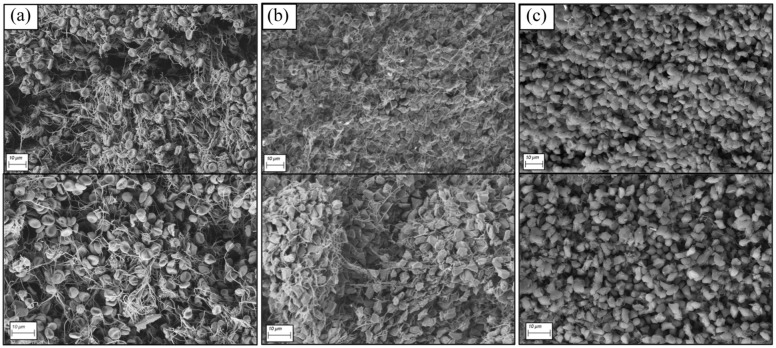
Representative SEM images of the RBC dense core region in (a) uncompacted 10 Hct EAs, (b) compacted 10 Hct EAs that have undergone simulated AIS with 125 mmHg proximal pressure, and (c) patient clots that were extracted by EVT.

SEM images showed the uncompacted 10 Hct EAs (Figure 5(a)) were primarily composed of loosely packed biconcave RBCs in a porous fibrin mesh. In contrast, the EAs that were compacted under 125 mmHg pressure showed a significant increase in RBC density (Figure 5(b)). The increased cellular component density corresponded with closure of the porous space as the gaps between RBCs became smaller. Most RBCs had transformed from biconcave disks into complete or intermediate polyhedrocytes with noticeable membrane morphological changes. In addition, the fibrin fibers appeared to be forced into the small gaps between polyhedrocytes. Like the histological results, the SEM images of the extracted patient clots (Figure 5(c)) were most like those of the compacted 10 Hct EAs. A similar fibrin fiber densification effect was also observed in SEM images of compacted 0 Hct EAs (Supplemental Figure SF5).

### Clot permeability measurements

**Figure 6.**
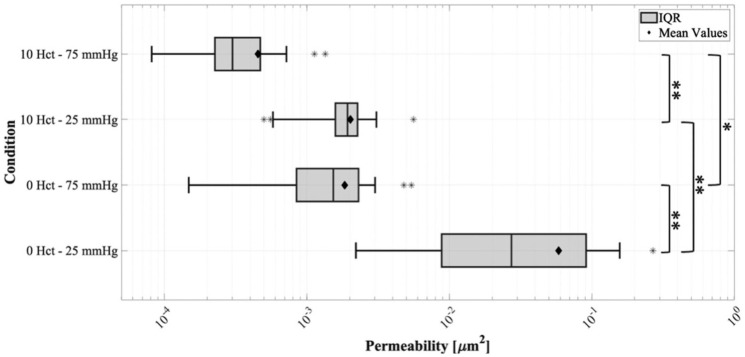
Permeability comparison between the 0 and 10 Hct clots under two different compaction pressures (*n* = 18 for each EA type and pressure). *p < 0.05. **p < 0.005.

Both EA types showed a significant permeability decrease (*p* < 0.005) as compaction pressure increased (Figure 6). The 10 Hct EA permeability decreased 85% from 1.9 × 10^−3^ µm^2^ (IQR: 1.6 × 10^−3^–2.3 × 10^−3^ µm^2^) under 25 mmHg pressure to 0.3 × 10^−3^ µm^2^ (IQR: 0.2 × 10^−3^–0.5 × 10^−3^ µm^2^) under 75 mmHg pressure. Similarly, the 0 Hct permeability decreased 95% from 27.3 × 10^−3^ µm^2^ (IQR: 8.8 × 10^−3^–91.1 × 10^−3^ µm^2^) to 1.5 × 10^−3^ µm^2^ (IQR: 0.8 × 10^−3^–2.3 × 10^−3^ µm^2^). The permeabilities for the 0 Hct clots were significantly higher than that of the 10 Hct clots under both 25 (*p* < 0.005) and 75 mmHg (*p* < 0.05) pressure.

## Discussion

This study is the first to incorporate in vitro embolization and distal vessel occlusion of EAs formed in environments intended to replicate in vivo thrombosis. Both fibrin/platelet dominant and RBC dominant EAs, representing two ends of the clinical AIS clot spectrum, are used for testing the novel mechanistic hypothesis proposed. Table 1 summarizes how our findings provide a physical explanation for several SVS positive clinical correlations. Our results conclude that RBC rich clots under high compaction forces during lodging are most likely to present as SVS positive and resist thrombolytic treatment. This conclusion aligns well with many of the clinical indicators that have been identified. Without consideration of mechanical compaction, it is difficult to reconcile how all the patient-specific factors and clinical outcome trends listed can be linked to SVS. Thus, existing clinical data supports our hypothesis.

**Table 1.** Summary of established SVS clinical correlations and indications along with mechanistic hypotheses that are supported by the results of this study.

Positive SVS clinical correlation	Ref.	Mechanistic hypothesis
Poor patient recovery/outcome	^22,23,60,61^	Higher density RBC clots compact into vessels, which reduces permeation and brain region perfusion
Lower early recanalization rate	^60,61^	Slower transport within the clot and higher RBC concentrations slow fibrinolytic protein binding and clot dissolution progression
High systolic blood pressure	^22^	Increased mechanical compaction force leads to clot compaction and elevated RBC concentration
Reduced vessel perfusion (TICI)	^22^	Lower clot permeability due to high RBC and polyhedrocyte concentrations
Poorer initial patient evaluation (NIHSS)	^22,61^	Reduced vessel perfusion because of the low permeability of compacted clots leads to higher infarct rate and final infarct volume
Cardioembolic stroke subtype	^62^	Low shear flow induced by atrial fibrillation or anatomical defects (i.e. atrial appendage) leads immature, soft, RBC rich clots that are more susceptible to compaction
Higher RBC and lower fibrin fractions	^63^	Soft clots with high RBC fraction are more deformable to squeeze into vessel, leading to higher RBC fractions
Larger clot length	^64^	Clots are extended axially in the vessel lumen as they are radially compacted into the smaller vessel
Increased symptom onset to imaging	^21^	Increased deoxyhemoglobin concentration in older clots with high RBC fractions

### Mechanical compaction during AIS causes clot microstructural changes that can be detected using MRI

The microscopy data show that microstructural changes in AIS clots can be driven by mechanical compaction during vessel occlusion. The degree of mechanical compaction is affected by both thromboembolism type and compaction pressure, which vary based on patient-specific factors including stroke etiology, systemic pressure, stroke location, proximal vessel anatomy, occlusion fraction, and cerebral collateral development status.^36^

In general, compaction leads to increased clot constituent density and reduced porosity. The porosity decreases by 72% and 81% in the 10 and 0 Hct EAs, respectively, compacted at 125 mmHg. The SEM images show sparsely distributed constituents and large pores in uncompacted EAs, whereas the compacted EAs have densely packed fibrin fiber bundles, RBCs, and polyhedrocytes with minimal interstitial porous space. The average R2 relaxation rate significantly increases following compaction, again indicating greater clot constituent density. The positive linear correlation between compaction pressure and R2 shows the densification effect is directly dependent on the applied pressure forces. The most significant R2 changes in the EA tissue occur in the distal region where the vessel is smallest due to the taper, which indicates there is a higher RBC and fibrin fiber fraction in this region during compaction. The smaller distal region is where the clot undergoes the most compaction, which would align with greater increases in solid constituent fractions. The observed decrease in EA volume as a function of applied pressure suggests that constituent densification results from interstitial porous space collapse as blood cells and fibrin fibers are compressed together, and the plateauing of this relationship beyond 100 mmHg suggests there is a compaction threshold. Similar results were observed for each clot type tested across six different donors, suggesting that the mechanisms underlying the microstructural and MR property changes are universal and supporting broader clinical translatability. To further support translatability, the proximal compaction pressures used in this study are clinically relevant. The mean pressure difference across an occlusive AIS clot is 63.1 ± 19.1 and 56.7 ± 18.0 mmHg among patients with poor and favorable functional outcomes, respectively.^28^ Therefore, the compaction pressures experienced by the EAs are like the pressures experienced by typical AIS clots in vivo and the experimental pressure range sweeps across the 95% confidence interval for clinical measures. A separate clinical study found the differences between the proximal and distal mean systolic and diastolic blood pressures were 71.4 and 26.7 mmHg, respectively.^29^ Again, demonstrating that our chosen experimental pressures are within the range of clinical values.

There were no uncompacted EAs observed in our study where platelet-mediated contraction alone generated polyhedrocytes, which may be due to the acute formation protocol or low thrombin concentration.^37^ However, the compaction forces generated on RBC dominant EAs during vessel occlusion were sufficient to form polyhedrocytes that appear similar to those formed by platelet-mediated clot contraction.^37–41^ This suggests that induced compaction during lodging is an additional mechanism for polyhedrocyte formation, which has not been previously reported. Polyhedrocyte formation explains why some cases had distal region hematocrit estimates above 100%. As RBCs pack together to form polyhedrocytes, the RBC concentration would begin to exceed that of the calibration samples.

### Compaction pressure influences MR relaxation rates dictating the presence of SVS

Increases to clot tissue R2 are clinically significant for AIS detection because higher R2 relaxation rates lead to signal hypointensity at the thrombus location when using T2-w or T2*-w pulse sequences, like SWI. A previous ex vivo study showed that the average R2 of human brain tissue in a 14 Tesla scanner is approximately 35 s^−1^.^42^ The R2 of the uncompacted 0 and 10 Hct EAs in our study were 10.0 and 15.7 s^−1^, respectively. Therefore, neither of the uncompacted EAs would appear hypointense in a T2-w image because the R2 rates are slower than the brain tissue. A short TE T2-w pulse sequence would even result in a hyperintense appearance for these uncompacted EAs. However, following compaction at 125 mmHg, the 0 and 10 Hct EAs had average R2 relaxation rates of 24.3 and 50.9 s^−1^, respectively. The compacted 0 Hct EA R2 rate remains lower than that of surrounding brain tissue and would still present as SVS negative, which is consistent with previous findings that SVS negative clots are often platelet/fibrin rich.^43^ Thus, shortened TE pulse sequences would be required to detect and distinguish these fibrin-dominant clots. The average R2 relaxation rate of the compacted 10 Hct EA is significantly higher than the surrounding brain tissue and would present as SVS positive. These results show that mechanical compaction due to thromboembolism lodging can contribute to the presence of SVS. The amount by which this SVS hypointensity shift occurs is dependent on the pressure drop across the clot and the original clot type.

### Clot compaction affects thrombolytic susceptibility

Our study shows that SVS positive clots can present because thrombus compaction during occlusion leads to elevated RBC density and R2 relaxation rates. The only samples that have R2 relaxation rates elevated above that of brain tissue and would present clinically with SVS were the highly compacted, RBC rich clots that have low porosity and polyhedrocytes present. Consequently, this clot type also had the lowest permeability and the largest occluded length, which suggests an increased resistance to IVT. Effective dissolution relies on adequate transport of the thrombolytic agent and endogenous fibrinolytic proteins through the clot.^44,45^ Clots with lower permeability are expected to have greater thrombolytic resistance because of the limited advective transport of dissolved proteins. In addition, closure of the porous space during compaction causes expulsion of interstitial serum containing endogenous fibrinolytic proteins.^46,47^ With the resultant reduction in internal fibrinolytic protein concentration, the dissolution process becomes even more dependent on external thrombolytic delivery, which is hindered by the permeability reduction. Thus, the same type of clot in a patient with average to poor cerebral collateral development and a higher occlusion pressure drop, like in the 75 mmHg compaction case used in the permeability experiments, would have a greater resistance to thrombolytics than the same occlusive clot in a patient with good collateral development and a lower pressure drop, like the 25 mmHg case. One final potential mechanism identified that supports the connection between pressure mediated compaction of RBC-rich clots and fibrinolytic resistance is the limited access to fibrin fiber surface area in the internal clot structure. Although not quantified in this study, the fibrin fibers in the SEM images of the compacted 10 Hct EAs, particularly in the clot core, appeared sparser. In some regions fibrin fibers were encapsulated between densely packed polyhedrocytes. This type of microstructural formation would make it increasingly difficult for fibrinolytic proteins to reach fibrin binding sites, slowing the dissolution rate.

The 0 Hct EAs had lower R2 relaxation rates than the 10 Hct EAs and did not surpass the SVS positive R2 threshold. However, there remains evidence that compaction influences their thrombolytic susceptibility. The increased R2 following compaction demonstrates increased fibrin volume fraction, that once again suggests elevated thrombolytic resistance through reduced permeability and elevated fibrin concentration.^48^ We also quantified the relaxation rate ratio (R2/R1) in the 0 Hct EAs because the formation and contraction of organic porous media, such as fibrin gels and blood clots, leads to an increase in *R2*/*R1*, that is correlated with elevated internal surface-to-volume ratios.^35^ A similar *R2*/*R1* trend was found in our data as the 0 Hct EAs underwent compaction, increasing from 27.9 in the uncompacted state to 55.2 at 125 mmHg pressure. Increased surface-to-volume ratio has interesting implications on the thrombolytic susceptibility because it suggests collapse of the interstitial porous space and maintained fiber surface area during compaction. Overall, these results indicate there is value in performing MRI-based clot characterization beyond binary SVS classification because it provides information on compaction state and thrombolytic susceptibility.

Current clinical MRI imaging techniques for AIS patients, such as SWI, are limited to a binary determination of SVS positive or negative. However, our study shows that expansion of MRI techniques toward quantifying relaxation rates of the clot tissue can provide detailed insights into the microstructure of the occlusive clot in vivo and aid in the development of a protocol for readily identifying IVT susceptible clots for more effective AIS patient treatment. In conclusion, this study demonstrates that mechanical compaction of thromboemboli during lodging may reduce the effectiveness of thrombolytic therapy for AIS by increasing the clot microstructural density, which can be detected using MRI.

### Study limitations and future directions

The elevated intra-donor variability of the RBC calibration samples was caused likely by the high sensitivity of the R2 relaxation rate to hemoglobin oxygenation.^49,50^ Individual donor and experimental factors, such as blood oxygen saturation and time from blood draw to MRI scan, directly impact R2. We did not account for the effects of intra-donor variability in blood oxygen saturation. Instead, we took a bulk averaging approach to develop a calibration curve that applies for hematocrit estimates across all donors.

The MRI scans under different compaction pressures were acquired for one 0 Hct EA and one 10 Hct EA from each donor under sequentially increasing pressures. We assume that the observed changes to EA relaxation rates and volumes are a result of the changing compaction pressure. However, clots are viscoelastic and will have a creep response to a constant applied stress. We mitigated these temporal effects by incorporating an equilibration time and minimizing scan time for each acquired pulse sequence. Previous studies exploring the mechanical properties of human blood clots under compression found the viscoelastic relaxation response plateaued at approximately 200 s, which suggests the 300 s equilibration period used in this study is sufficient to neglect most creep effects.^51–55^ Motion artifacts were not observable near the clot boundaries in any of our scans, further indicating that the equilibration period was effective. Therefore, although time dependent deformation remains a potential confounding factor, compaction pressure was the dominant factor associated with R2 increase and volume reduction.

The MRI and permeability experiments capture the acute occlusion event and fluid permeation under steady pressures, whereas AIS clots in patients undergo a prolonged occlusion event with time-varying pressure forces. These temporal effects occur on both short timescales (i.e. cardiac pulsation) and long timescales (i.e. systemic blood pressure regulation), which will add to the complexity of predicting microstructural compaction compared to this in vitro model. An additional consideration is that thrombolytic perfusion through the clot will also be dependent on the complex surrounding hemodynamic environment in vivo. Changing hemodynamics will continuously alter the clot microstructure, which means the clot permeability will likely be time-dependent and not a constant value as the permeability experiments suggest.

All experiments are performed only with two EA types that do not represent the broad spectrum of mixed, heterogeneous clinical thrombi (Figure 4(a)),^56–58^ despite using physiological coagulation factor concentrations and dynamic Chandler Loop formation conditions. Therefore, results demonstrate that the clot compaction effects are present for both RBC and fibrin/platelet dominant clots but there is limited knowledge about the effects on realistic mixed thrombi.

The AIS simulations may not recapitulate accurately embolus motion because the frictional forces between the clot and endothelium in vivo differ from those between the EA and MCA mimicking channel in vitro. The frictional forces between the clot and the 3D printed channel are expected to be higher than in vivo due to the greater surface roughness compared to a healthy endothelium. This would result in our experiments underpredicting the compaction effect magnitude because the EAs would reach equilibrium at a more proximal location.

The focus of this study is limited to the acute compaction effects on the MR relaxation rates and did not consider the effects of clot aging following occlusion that leads to hemoglobin deoxygenation. These changes would further amplify the local hypointensity shift, particularly in clinical SWI scans of RBC rich clots. Therefore, SWI signal intensities are sensitive to both the acute compaction effect observed in this study and time-dependent aging effects. These effects occurring simultaneously will make it more challenging to discern which is the dominant factor contributing to the presence of SVS in clinical scans.

Although we were able to compare EA microstructure with extracted patient clots, the patient clots could not be scanned in the 14 Tesla MRI for comparison with the EAs because the extracted clots were received after being fixed in PFA for an extended period. The fixation and aging significantly alters the MR properties of the tissue, which precludes direct comparison with the fresh EAs.

To improve clinical translatability, future work should focus on expanding this experimental platform toward clinical conditions. This includes using excised vessels or endothelial cell lined tubes as the MCA mimicking channel to better replicate the frictional and interaction forces. EA formation methods should be expanded to include a broader spectrum of mixed clot types by altering the initial blood reconstitution and Chandler Loop shear rates, which will verify that this compaction mechanism is observed universally.^59^ Finally, time-varying compaction pressures should be applied to observe how realistic hemodynamic changes may alter the observed effects on clot microstructure, MR relaxation rates, permeability, and thrombolytic susceptibility.

## Non-standard abbreviations and acronyms

AIS Acute ischemic stroke

EA Embolus analog

EVT Endovascular thrombectomy

IVT Intravenous thrombolytic therapy

LVO Large vessel occlusion

MCA Middle cerebral artery

MRI Magnetic resonance imaging

ROI Region of interest

PPP Platelet poor plasma

PRP Platelet rich plasma

RBC Red blood cell

SEM Scanning electron microscopy

SVS Susceptibility vessel sign

SWI Susceptibility weighted imaging

## Supplemental Material

sj-docx-1-jcb-10.1177_0271678X261465841 – Supplemental material for Mechanical compaction alters microstructural and magnetic resonance imaging properties of acute ischemic stroke clots Supplemental material, sj-docx-1-jcb-10.1177_0271678X261465841 for Mechanical compaction alters microstructural and magnetic resonance imaging properties of acute ischemic stroke clots by Cody J Kubicki, Thomas Neuberger, Scott D Simon and Keefe B Manning in Journal of Cerebral Blood Flow & Metabolism
