# Supplementary material for: Mechanical compaction alters microstructural and magnetic resonance imaging properties of acute ischemic stroke clots
Source: J Cereb Blood Flow Metab. 2026 Jun 25:0271678X261465841. Online ahead of print. doi: 10.1177/0271678X261465841 (PMC13354588; doi:10.1177/0271678X261465841)
Supplement: sj-docx-1-jcb-10.1177_0271678X261465841 – Supplemental material for Mechanical compaction alters microstructural and magnetic resonance imaging properties of acute ischemic stroke clots [file sj-docx-1-jcb-10.1177_0271678X261465841.docx]

**Title:** Mechanical Compaction Alters Microstructural and Magnetic Resonance Imaging Properties of Acute Ischemic Stroke Clots – Supplemental Material

**Authors:** Cody J. Kubicki^1^, Thomas Neuberger^2^, Scott D. Simon^3^, Keefe B. Manning^1,4^

^1^ Department of Biomedical Engineering, The Pennsylvania State University, University Park, PA, United States

^2^ Huck Institutes of Life Science, The Pennsylvania State University, University Park, PA, United States

^3^ Department of Neurosurgery, Penn State College of Medicine, Hershey, PA, United States

^4^ Department of Surgery, Penn State College of Medicine, Hershey, PA, United States

**Supplemental Figures**

**
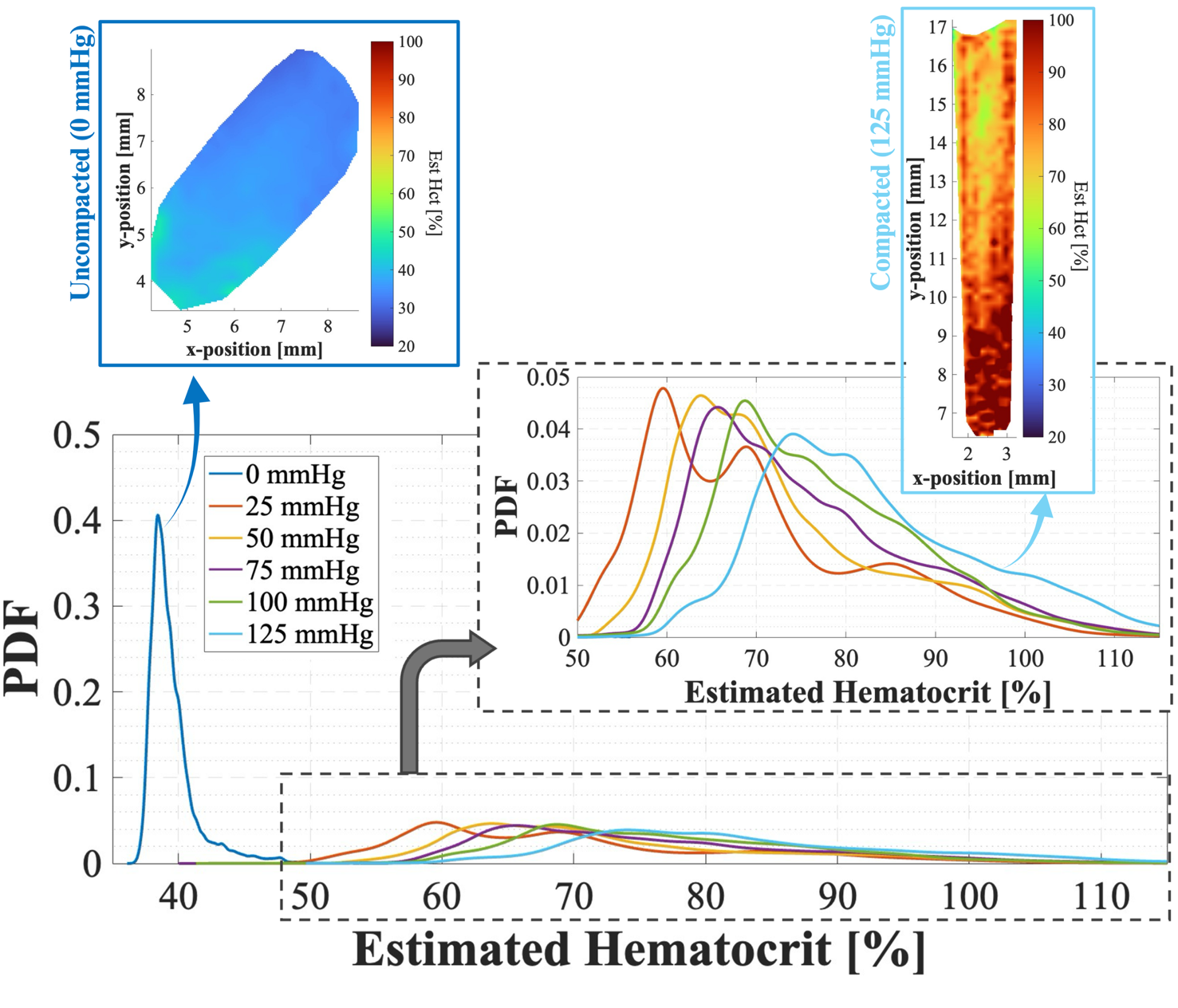
**

**Supplemental Figure SF1:** Probability density function (PDF) distribution curves of the pixel-wise estimated hematocrit within the clot tissue ROI for a second representative 10 Hct sample under all six compaction pressure conditions that shows an example case where some regions had hematocrit estimates above 100%. The inset PDF plot shows the same plot focused on only the compaction cases to highlight the smaller differences between the compaction pressure conditions. The overlaid contour plots show the estimated hematocrit spatial distributions of the two respective conditions indicated (0 mmHg and 125 mmHg).

**
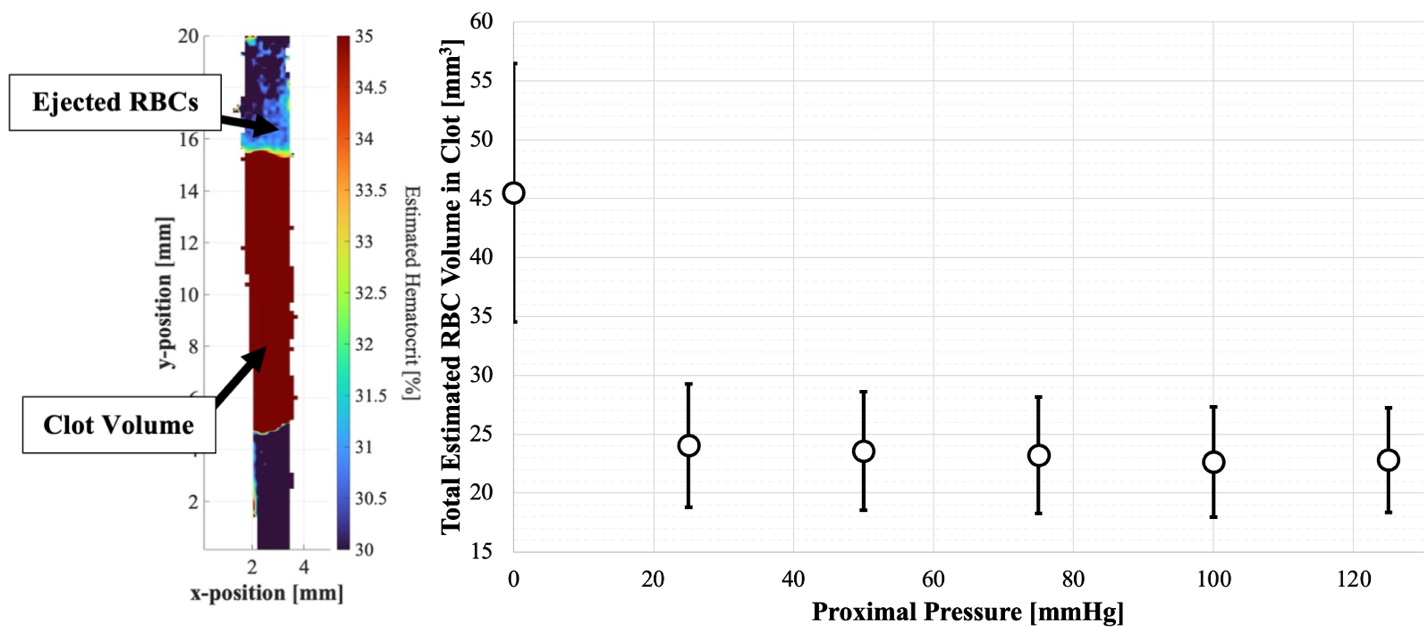
**

**Supplemental Figure SF2:** (A) Spatial map of estimated hematocrit (10 Hct sample under 125 mmHg pressure) in the full MRI FOV including regions in the MCA mimicking channel proximal and distal to the clot that shows the ejection of RBCs near the proximal clot face to a greater extent compared to the distal end of the clot. (B) Scatter plot of the total estimated clot volume occupied by RBCs as a function of compaction pressure shows that there is a significant reduction in total RBC volume following compaction, which corresponds to the ejection into the surrounding saline seen in the spatial map. Error bars represent the standard deviation.

**
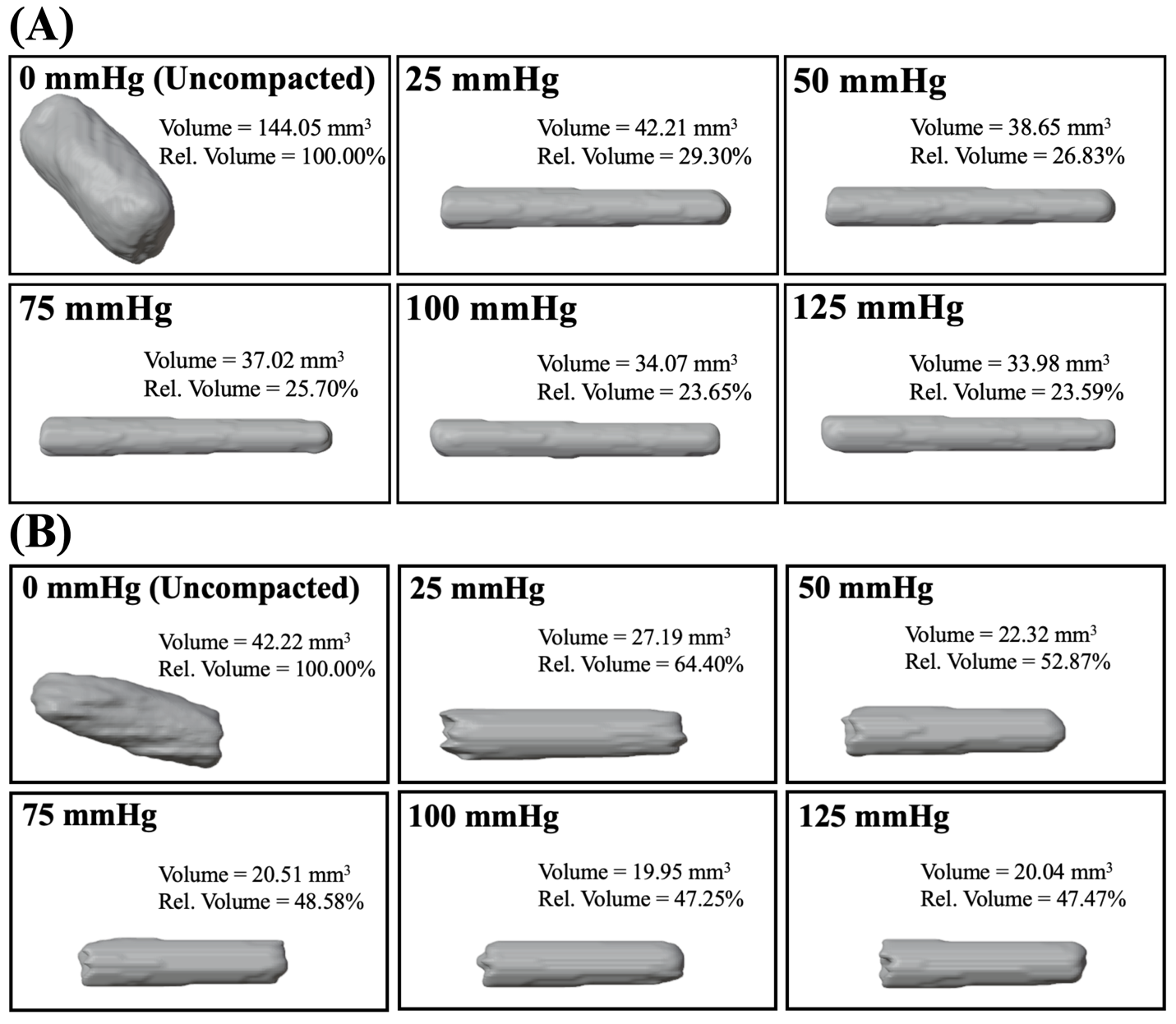
**

**Supplemental Figure SF3:** Representative 3D reconstructions of a (A) 10 Hct and (B) 0 Hct EA generated from the 3D-FLASH scans under each compaction pressure, along with their respective calculated absolute volume and volume relative to the initial uncompacted state. The reconstructions show the morphology changing to match the shape of the tapered vessel as the EA is pushed further distally under increasing pressures. Note that the spatial scales are different between the reconstructions of the two EA types.

**
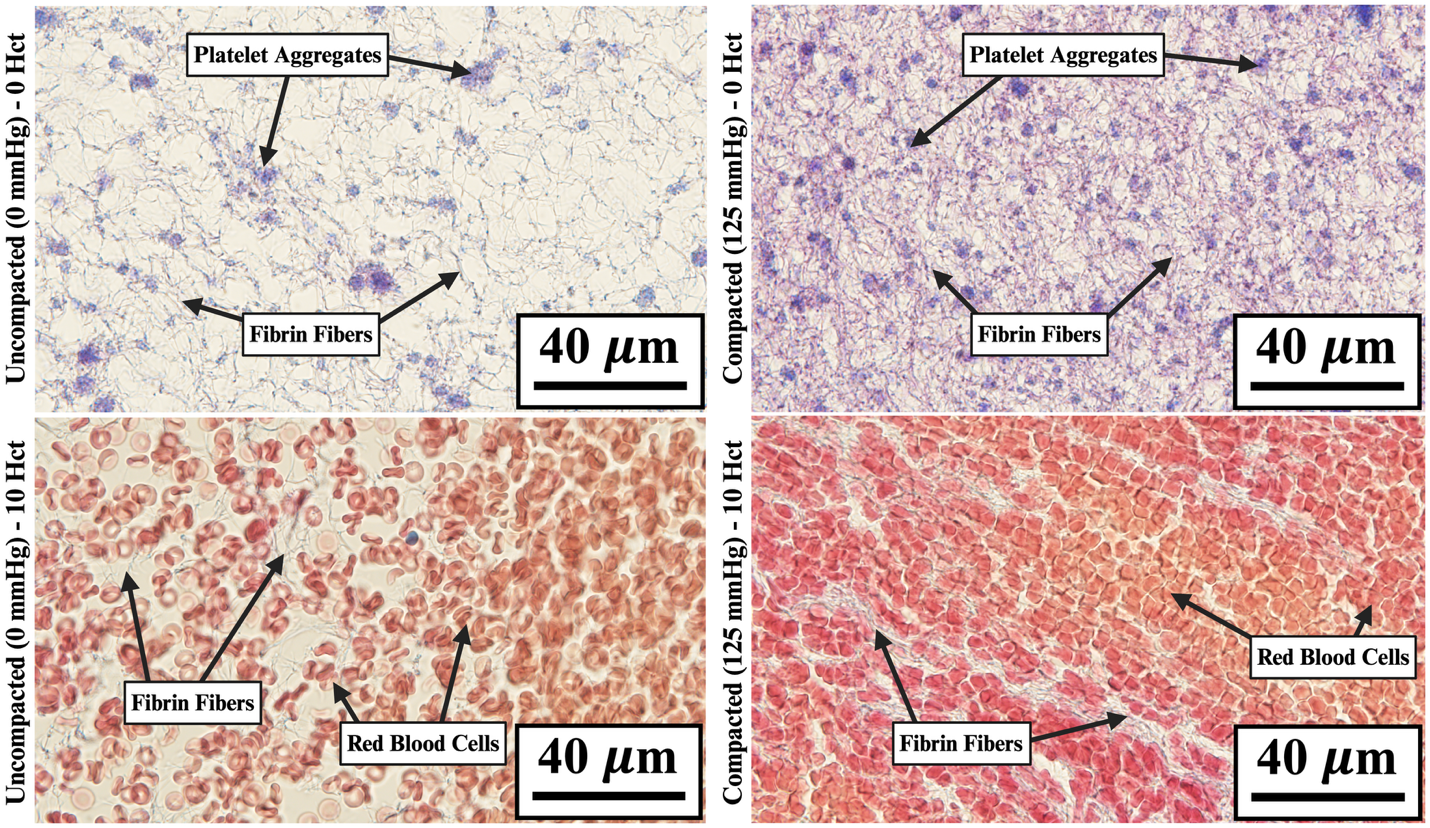
**

**Supplemental Figure SF4:** Representative high magnification (100x objective lens) images acquired for each of the clot types (0 and 10 Hct) at both the uncompacted (0 mmHg) and maximum compaction (125 mmHg) states to highlight the individual constituents. Formed element density increase is apparent in both clot types undergoing compaction, and RBC membrane morphology changes can be observed as the 10 Hct clot undergoes compaction and the RBCs transform to polyhedrocytes.

**
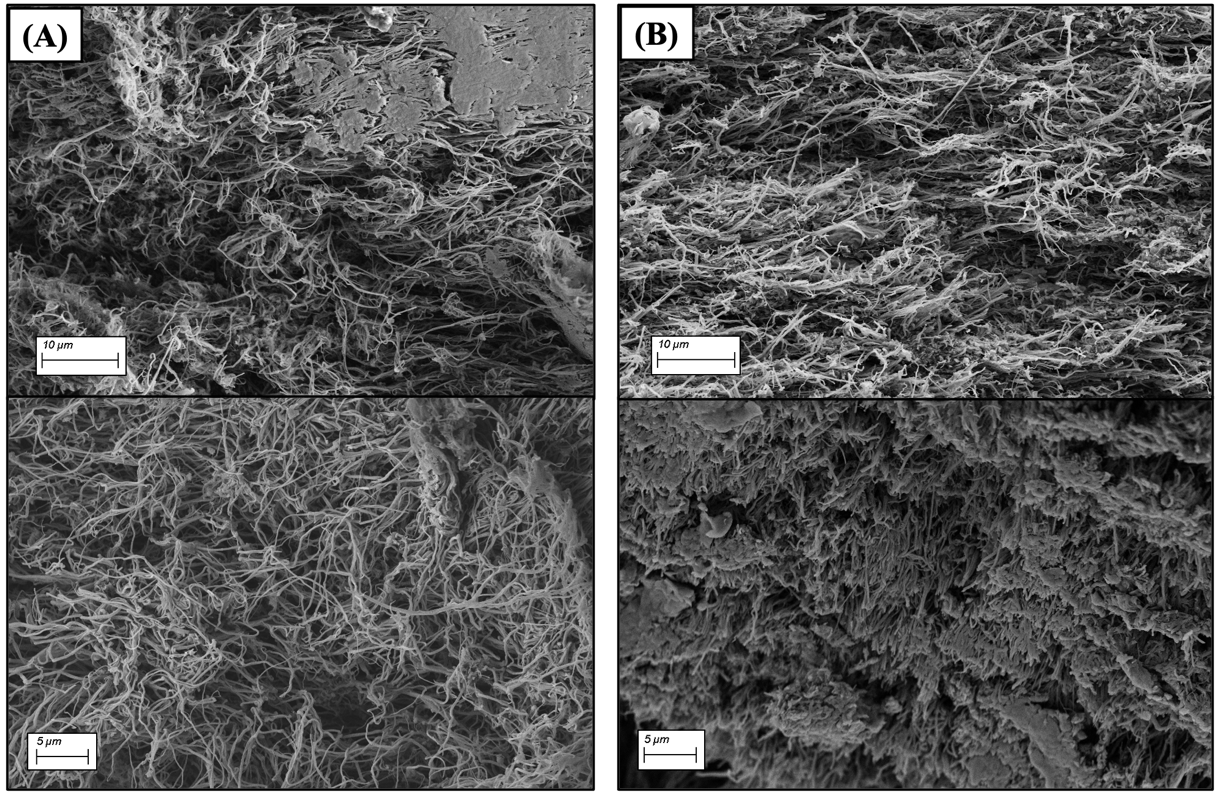
**

**Supplemental Figure SF5:** Representative SEM images of (A) uncompacted 0 Hct EAs and (B) compacted 0 Hct EAs at low and high magnifications that have undergone simulated AIS with 125 mmHg proximal pressure.

**Supplemental Tables**

**Supplemental Table ST1:** RARE-VTR and 3D-FLASH pulse sequence parameters used to acquire MRI data, including MR relaxation rates and volumetric reconstructions of EAs.

**
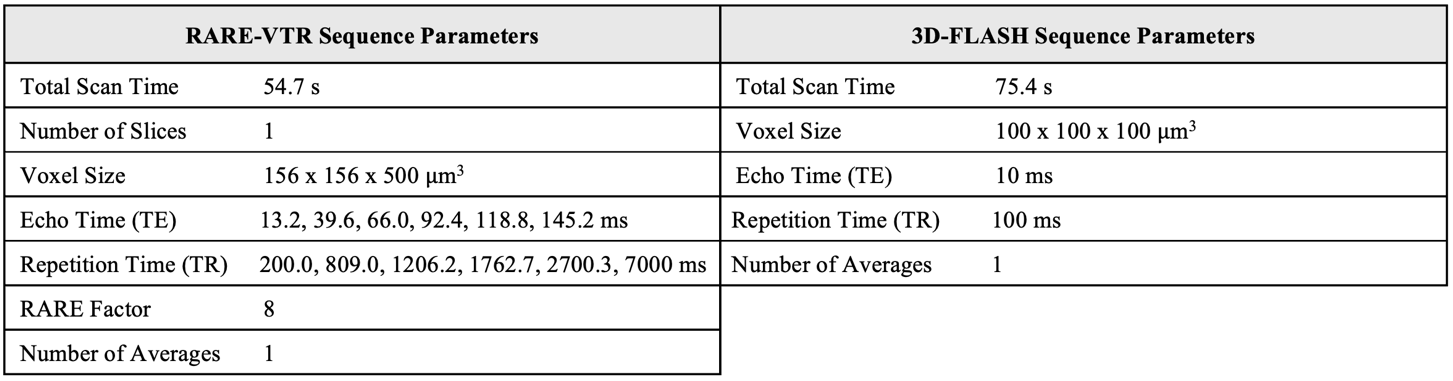
**

**Supplemental Table ST2:** Donor demographic summary for MRI experiments


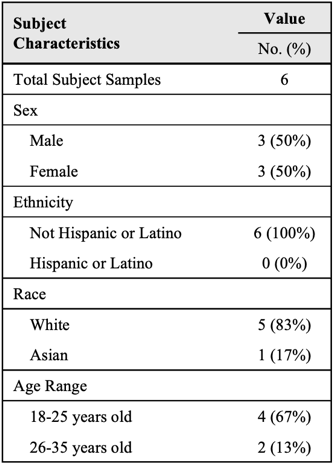


**Supplemental Table ST3:** Donor demographic summary for permeability quantification experiments


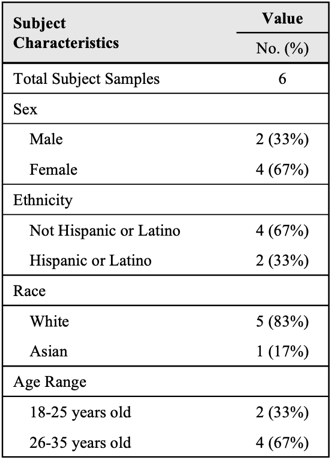


**Supplemental Table ST4:** Summary of patient demographic data for extracted samples used for imaging

**
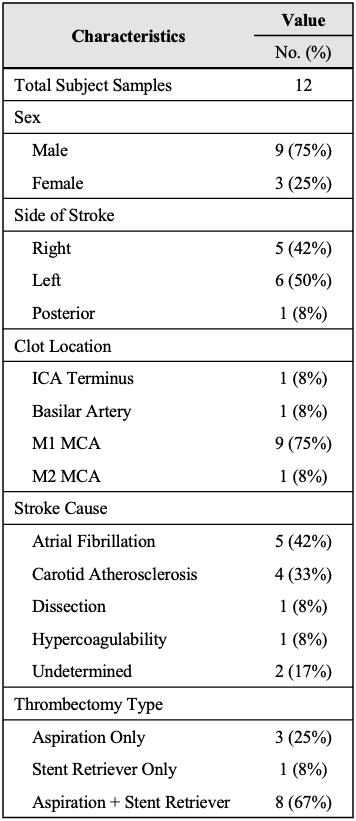
**

**Supplemental Table ST5:** Summary of the histological porosity estimates for compacted and uncompacted EAs and extracted patient clots (SD = standard deviation). The p-values shown represent comparisons between the respective EA type and the patient clot data.


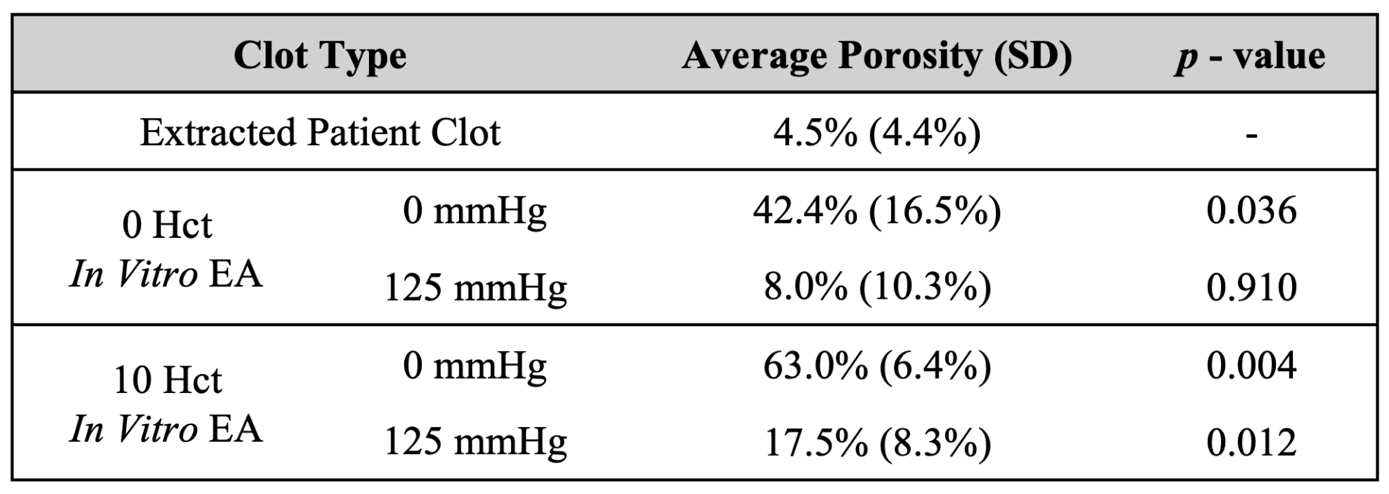


**Supplemental Methods SM1:** Magnetic Resonance Relaxation Rate Processing Details

*R2 Relaxation Rate Calibration Curve Fitting Details*

The average R2 relaxation rate within each calibration sample vial ROI was quantified using an in-house MATLAB processing script that calculated the pixel-wise R2 maps by performing signal intensity curve fitting across six images taken with different TE values at the longest TR (7000 ms) (**Fig. 1A**). The exponential decay curve (**Eqn. 1**) was used as the fit model to calculate the R2 value of each pixel using a linear least-squares regression method and trust-region-reflective algorithm.

| $S_{p,i}=M_{0}e^{-{TE}_{i}*R2_{p}}$ | (1) |
| --- | --- |

Where $M_{0}$ represents the equilibrium magnetization, ${TE}_{i}$ the echo time for scan *i* (*i =* 1, 2, …, 6), $S_{p,i}$ the signal intensity at pixel *p* for scan *i*, and ${R2}_{p}$ the transverse relaxation rate for pixel *p*. This process was repeated for each of the six donors used in the MRI experiments to generate a calibration curve relating average R2 in the 14 Tesla scanner to RBC fraction and plasma constituent concentration (**Fig. 1B**).

**
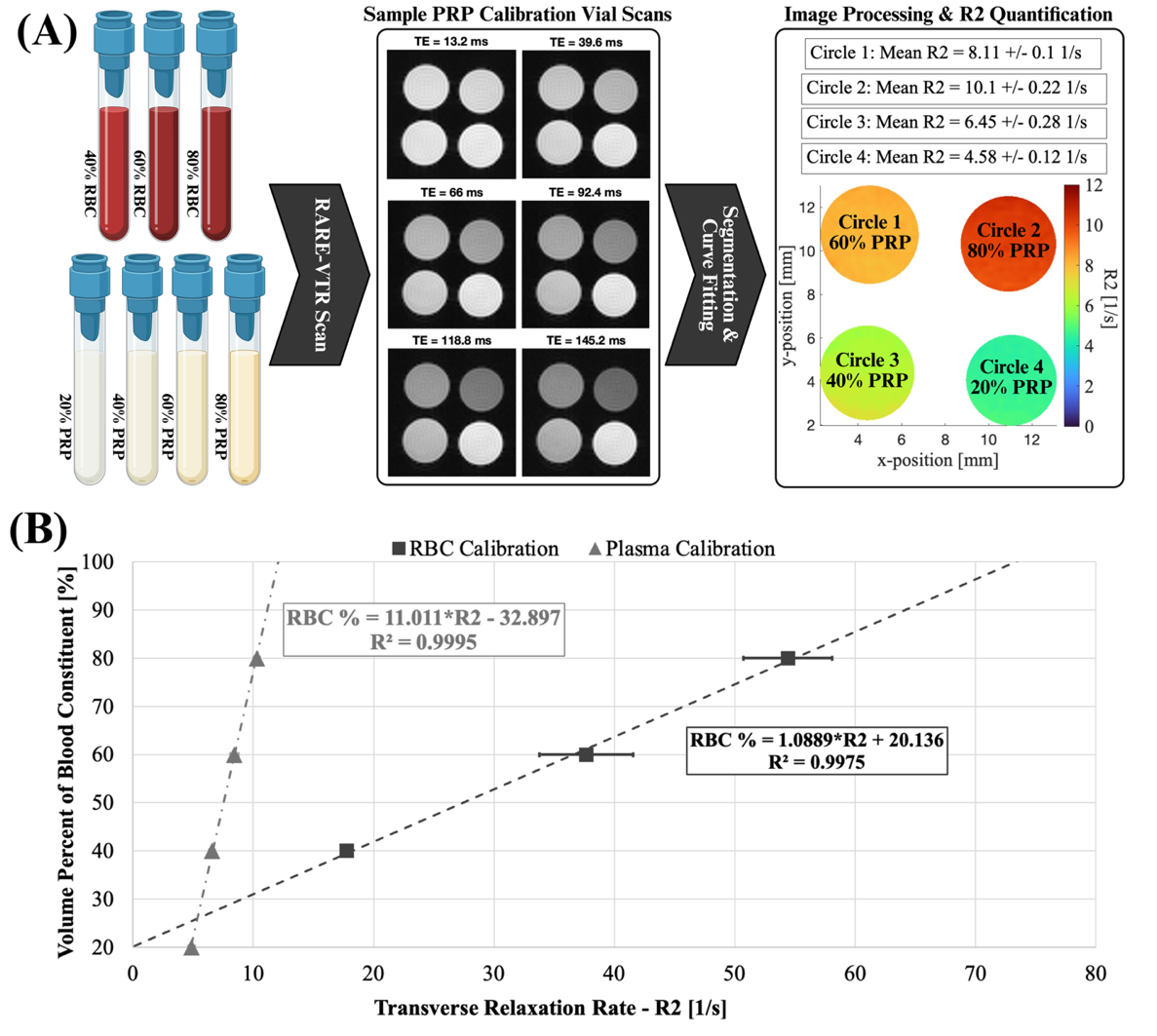
**

**Figure 1:** (A) Summary of the methodology used to calculate the R2 calibration curves for both RBC and plasma volume fractions in the 14 Tesla scanner. The middle panel shows representative scans of four plasma calibration vials. The right panel shows the corresponding R2 maps within each vial ROI following data processing and curve fitting. (B) Calibration curves for RBC and plasma component volume fractions related to the average R2 relaxation rate of the MR signal in calibration samples. Error bars represent the standard error of the mean.

*R1 and R2 Relaxation Rate Quantification in EA Samples*

The pixel-wise R2 and R1 relaxation rates were calculated from the RARE-VTR scans of the EAs using the same in-house MATLAB script described previously. Here, the processing code used the signal intensity at each pixel within a manually specified ROI capturing the entire EA across images taken with six different TE and TR values to perform curve-fitting to the transverse and longitudinal relaxation curves (**Eqn. 1** and **Eqn. 2**) to estimate the pixel-wise R2 and R1 relaxation rates, respectively.

| $S_{p,j}=M_{0}(1-e^{-{TR}_{j}*{R1}_{p}}$) | (2) |
| --- | --- |

Where $M_{0}$ represents the equilibrium magnetization for the material, ${TR}_{j}$ the repetition time for scan *j* (*j =* 1, 2, …, 6), $S_{p,j}$ the signal intensity at pixel *p* for scan *j*, and ${R1}_{p}$ the longitudinal relaxation rate for pixel *p*. R2 curve fitting was performed on images taken at the maximum TR (7000 ms) with varying TE, while R1 curve fitting was performed on images taken at the minimum TE (13.2 ms) with varying TR to maximize the recorded signal magnitude across all scans and optimize the curve fitting results. Any pixel locations that produced a poor fit (R^2^ < 0.9) during curve fitting were excluded from the dataset.

**Supplemental Methods SM2:** Standard Operating Procedure for Clot Constituent and Porosity Quantification Using Orbit Image Analysis

**Background and Sample Information:** Orbit Image Analysis is an open-source tissue that uses machine learning to classify/quantify tissue constituents. Orbit Image Analysis can be freely downloaded from <https://www.orbit.bio/download/> and is available for Windows, Mac, and Linux systems. Histology clot samples were sliced into 6 µm thick sections and stained using the Carstairs’ staining protocol to distinguish the primary clot constituents. Images of the stained samples were acquired using CellSens software connected to an Olympus BX61 microscope under brightfield with a 20x objective lens. The acquired images were then exported into .tif format to be transferred and processed in Orbit Image Analysis.

**Standard Operating Procedure:**

1. **Opening software**
   1. Following download and installation of the Orbit Image Analysis (OIA) software, open the software from the desktop icon (**Fig. 1**) or applications folder. (Note: initial pop-ups can be clicked through without setting up the image server)

**
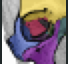
**

**Figure 1:** Orbit Image Analysis icon image


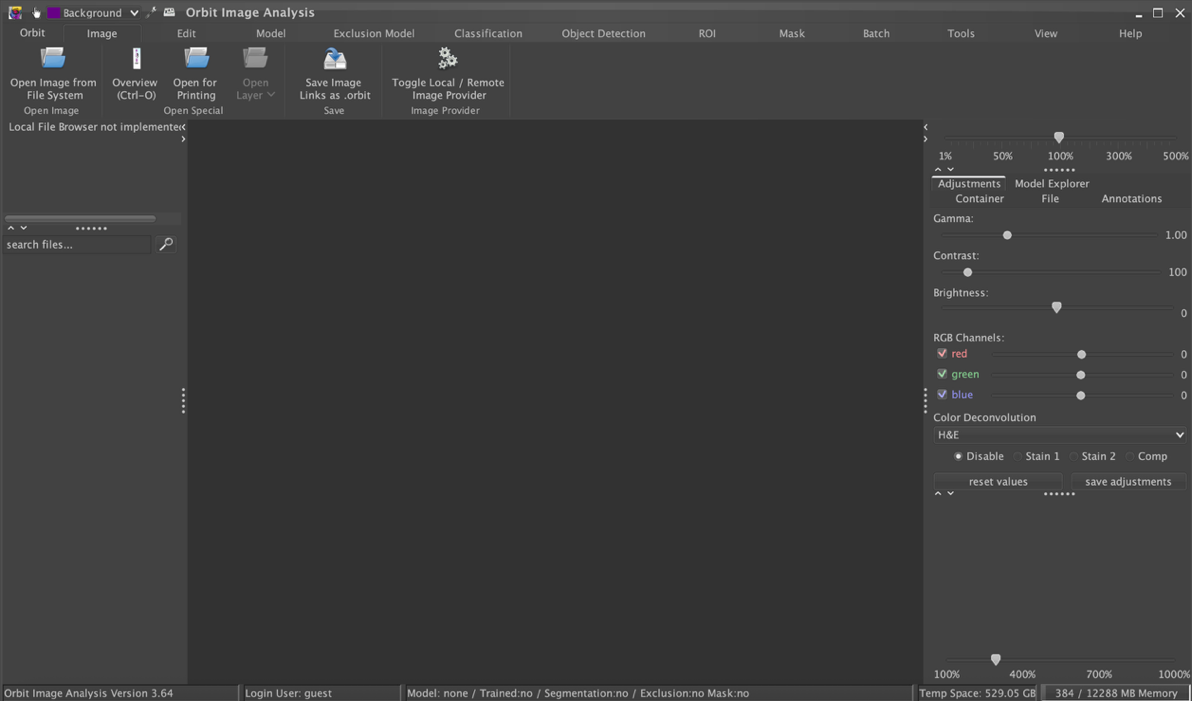


**Figure 2 :** Orbit Image Analysis user interface configuration

1. **Loading Classification Training Images**
   1. Open 3-4 images that are representative of the range of samples being processed and contain all the relevant constituents that you want to quantify
   2. Images can be opened/loaded into OIA by clicking “Open Image from File System” in the “Image” tab and then navigating to the directory containing your images, selecting them, and clicking “Open” or by dragging the image into the blank center viewing region of the OIA window

**
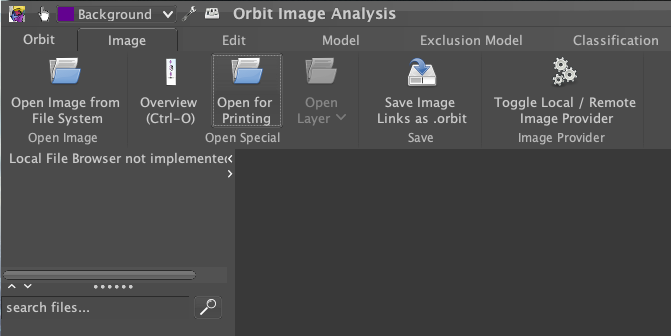
**

**Figure 3:** Loading images into OIA


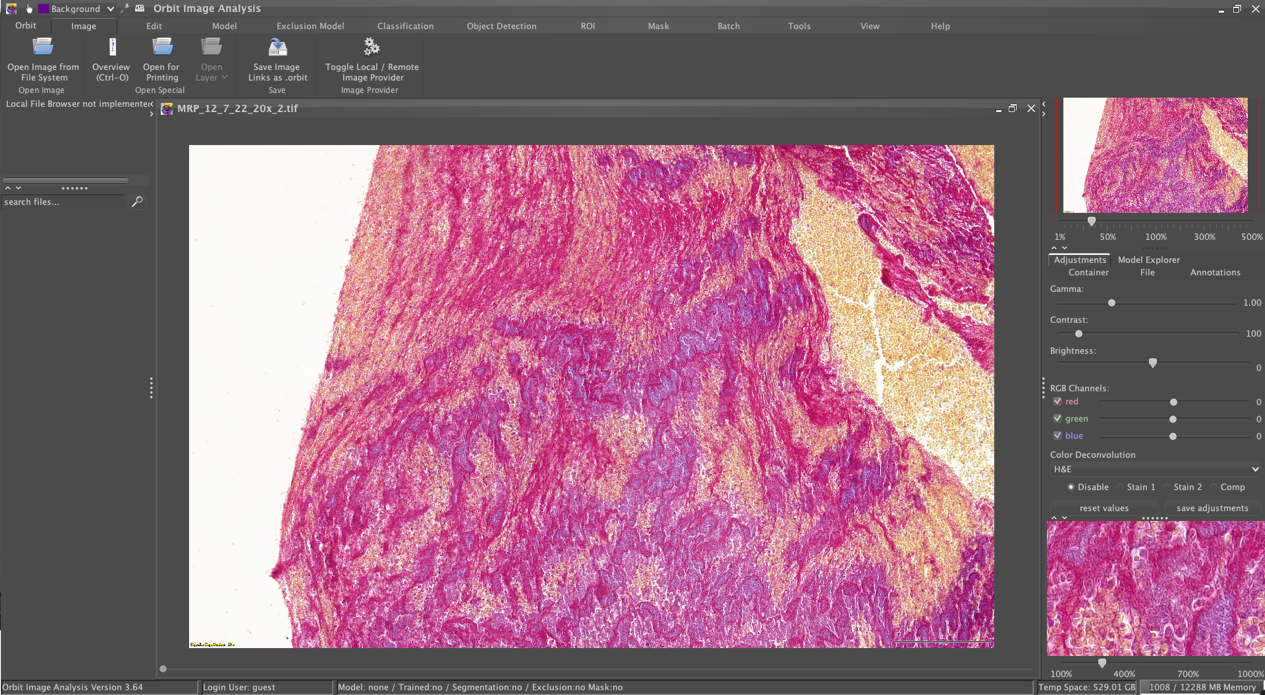


**Figure 4:** OIA user interface example following image loading

1. **Defining Clot Constituent Classes & Feature Configuration**
   1. Select the wrench icon in the upper-left corner of the main OIA window to open the “Classes” window (also found in the “Model” tab of the window)

**
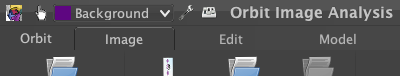
**

**Figure 5:** Icon to select for modifying classes included in model

- 1. Modify the color, name, and exclusion criteria for all the relevant cell types that you want to quantify, as well as for additional background and artifact (i.e., folding, image annotations/scale bars, etc.) classes. The color corresponds to the color that the classified pixels will appear as following identification based on the classification model. **Fig. 6** shows the setup used in the current study to classify all the tissue constituents stained using the Carstairs’ protocol (red blood cells, platelets, fibrin, and collagen). The background and artifacts are set to “Exclusion”, and all the other cell types are set to “Inclusion” to identify which constituents should be quantified if you want to use an exclusion model.

**
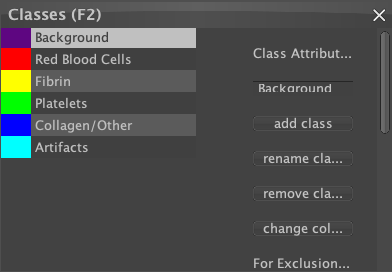
**

**Figure 6:** Class setup used for Carstairs’ staining protocol of blood clots

- 1. Once all the classes have been properly set up, scroll to the bottom of the Classes window and select “OK”
  2. Select the control sliders icon to the right of the wrench icon in the upper-left corner of the main OIA window to open the “Feature Configuration” window (also found in the “Model” tab of the window)

**
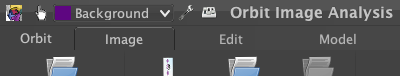
**

**Figure 7:** Icon to select for modifying feature configurations in the model

- 1. Set the “Structure size” to 1 and “Median filter radius” to 0. This will eliminate pixel smearing effects due to averaging and filtering, which allows the model to pick up small groups of background pixels right next to cell components in the interstitial to permit porosity estimation.

**
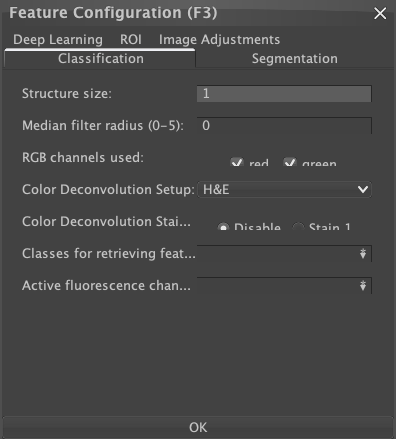
**

**Figure 8:** Feature configuration settings used in the current study – constituent and porosity quantification

1. **Drawing Regions of Interest (ROI) for Model Training**
   1. Select one of your classes in the dropdown menu in the top left of the main OIA window
   2. Draw multiple (4-5) ROIs around regions that contain the selected constituent type using the “Polygon”, “Circle”, and “Rectangle” ROI drawing tools. The “Eraser” tool can be used to remove unwanted ROIs from the image. The ROI boundary colors will match the representative color for the constituent comprising the ROI. Try to create ROIs in regions that are close to boundaries with other constituents without having any crossover of constituents to prevent the model from being able to distinguish between them.
   3. Repeat this process for all the classes included in the model. An example of a final set of ROIs for classification training on a single image is shown in **Fig. 9**.

**
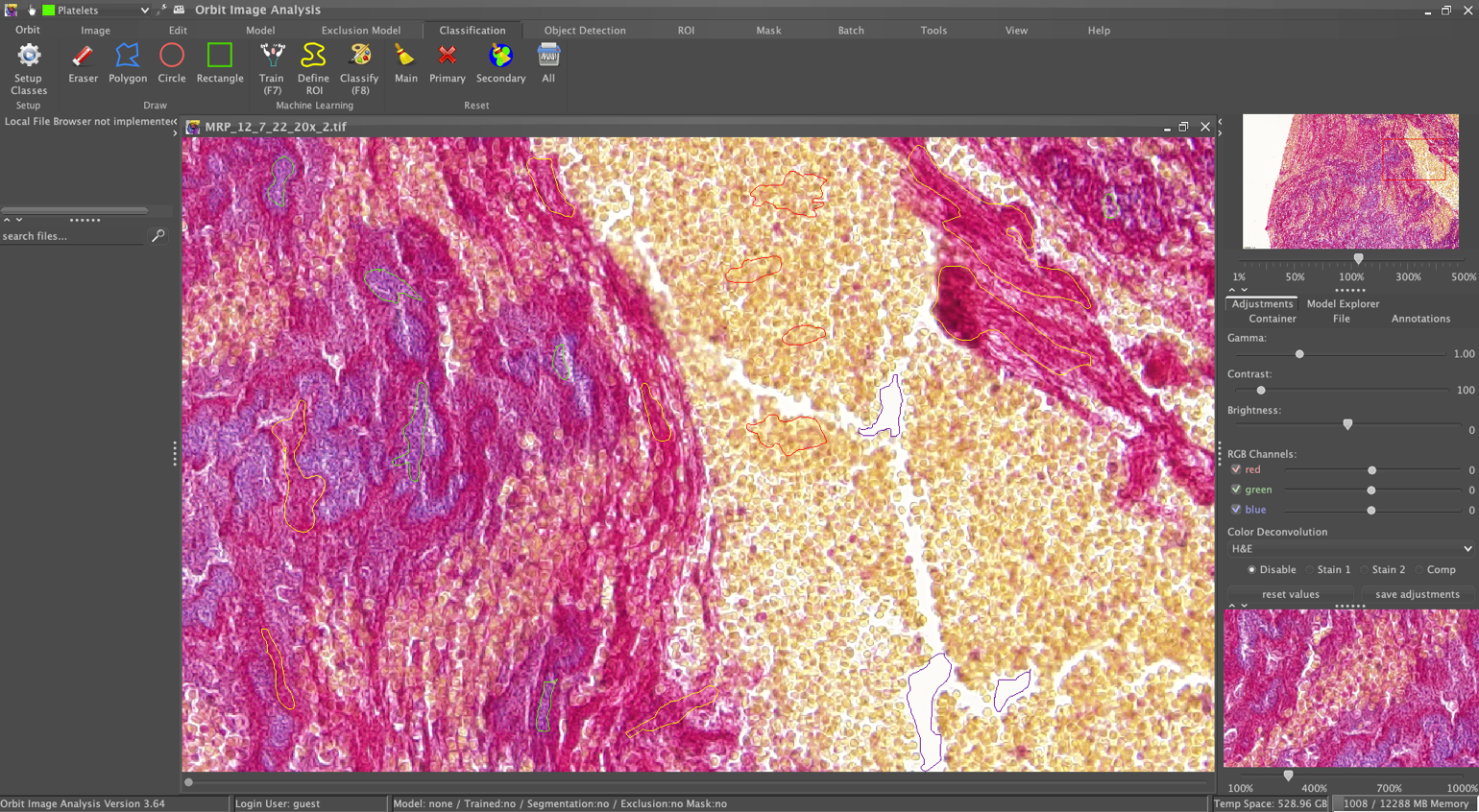
**

**Figure 9:** Example of ROIs drawn on a clot image used for classification training. The ROI drawing tools are highlighted by the white box and the dropdown menu used to select the class for each respective ROI highlighted by the white arrow.

- 1. Repeat this process with at least 2 other images, making sure to keep all the images loaded in the same window. Do not clear the ROIs until the training process is complete. This can be done by minimizing the image and moving to the next, or by making all the images viewable at once in the main window by selecting “Tile Windows” in the “View” tab

1. **Training the Classification Model:**
   1. Once you are satisfied with all the ROIs that will be used to train your classification model, go into the “Classification” tab and click the “Train” button.

**
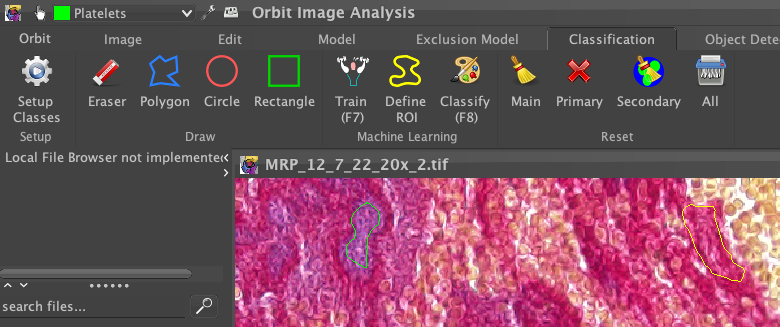
**

**Figure 10:** Training the classification model from the defined ROIs.

- 1. A loading bar will appear on the right-hand side of the window as the classification model is being trained. Once completed, the loading bar will disappear and the ribbon at the bottom of the window will update the training status and will now say “Trained: yes”.


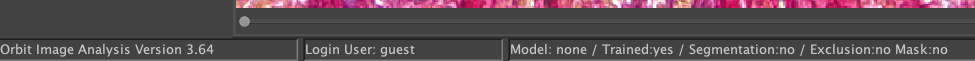


**Figure 11:** Training status update in the bottom ribbon to confirm model training completion.

- 1. Once the model has been trained, it can be saved for future use by selecting “Orbit” > “Save Model” > “Save Model as…”
     1. Saved models can be re-opened by selecting “Orbit” > “Open Model” > “Open Model” and navigating to select the previously saved model.

1. **Verifying the Trained Model**
   1. Select “Classify” in the “Classification” tab of the main OIA window. This will run the classification model on all the pixels of the currently selected image.

**
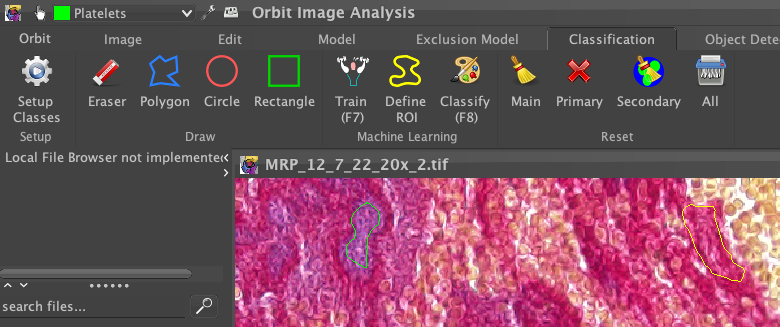
**

**Figure 12:** Running the classification model

- 1. A loading bar will appear to the right of the image that shows the progress of the classification model. Once classification is complete, the loading bar will disappear and an additional pop-up window for “Classification Results” will appear. Close out of the “Classification Result” window.
  2. Adjust the slider on the bottom of the image to change the opacity of the spatial overlay for the pixel-wise constituent classifications (**Fig. 13**). Pan around the image and adjust the slider to see both the raw image and the image with the constituent overlay visible. Verify that the classifications generally match the constituent patterns observed in the raw image across multiple clot locations and for multiple clot sample images.

**
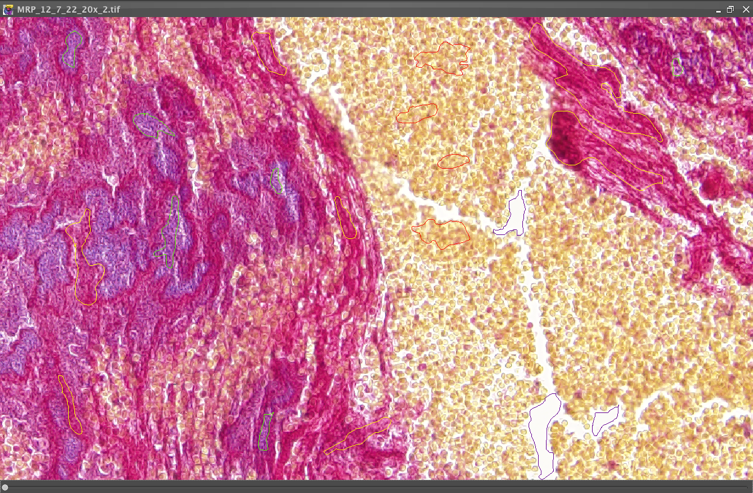

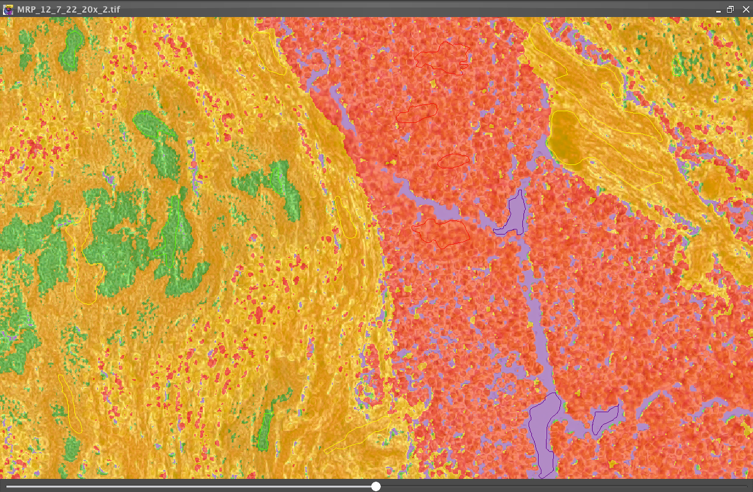
**

**Figure 13:** Adjusting the slider at the bottom of the image (white arrow) to increase the opacity of the constituent mapping overlay for model verification.

1. **Defining the ROI in Sample Images:**
   1. Click the “Define ROI” button in the “Classification” or “ROI” tab. The mouse cursor will change to crosshairs when you hover over the image once this is selected.

**
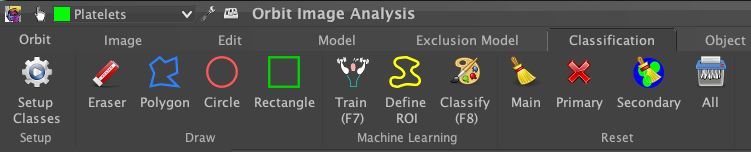
**

**Figure 14:** Icon to select to start drawing the ROI for tissue classification.

- 1. Draw the ROI by clicking and dragging across the image and releasing once finished (**Fig. 15**). Only what is inside the final ROI will be classified and quantified. For accurate porosity estimates that are not inflated due to histology slicing errors, only keep intact parts of the clot within the ROI. The only background pixels present in the ROI should be a result of interstitial space. Do not include any background pixels that are present because they are outside the clot boundary or because of sample tearing during the slicing and mounting processes.


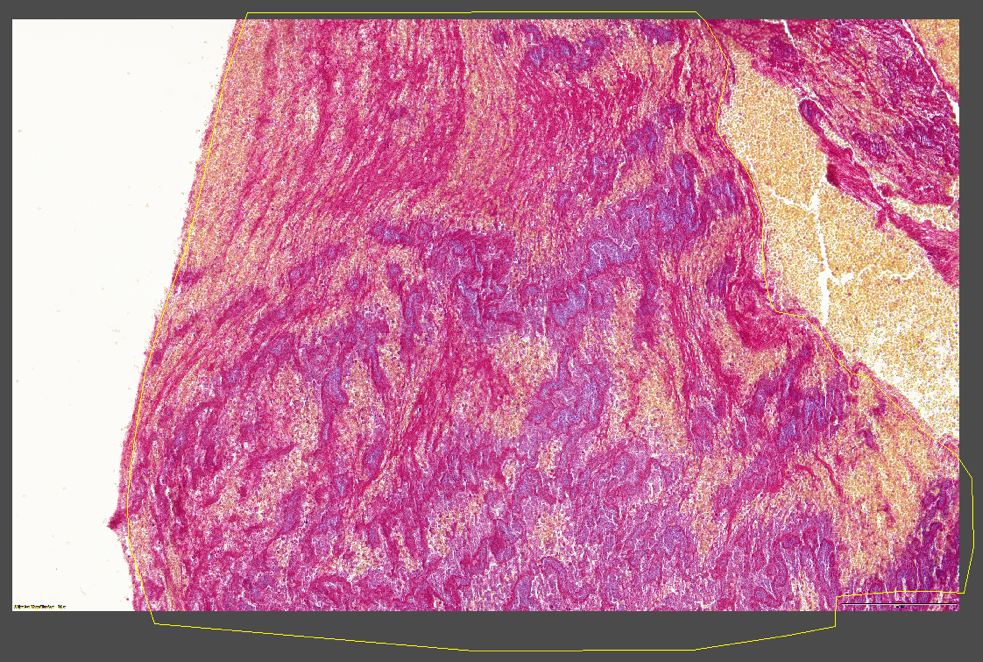


**Figure 15:** Example ROI (yellow line) defined for sample clot image. Black arrows highlight examples of regions outside the clot boundary and sample tears that should not be included in the final ROI.

1. **Running Classification Model and Quantifying Constituents:**
   1. Select “Classify” in the “Classification” tab of the main OIA window.
   2. A loading bar will appear to the right of the image that shows the progress of the classification model. Once classification is complete, the loading bar will disappear and an additional pop-up window for “Classification Results” will appear that quantifies the relative fractions of each of the constituents defined within the model (**Fig. 16**).
      1. The surface fractions of the constituents and background (interstitial space/porosity) can be converted into percentages by multiplying by 100.
   3. Record these results into a separate data processing software (i.e., Excel, MATLAB, etc.) to compile data for all the clot samples and perform additional post-processing.

**
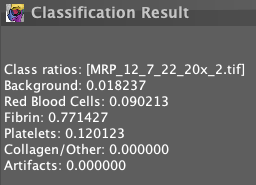
**

**Figure 16:** Classification results pop-up window detailing the quantitative classification results for the relative area fractions of each constituent within the ROI.

- 1. The slider below the image can again be adjusted to see the spatial map of constituents strictly within the selected ROI that corresponds to the quantitative results output by the model (**Fig. 17**).

**
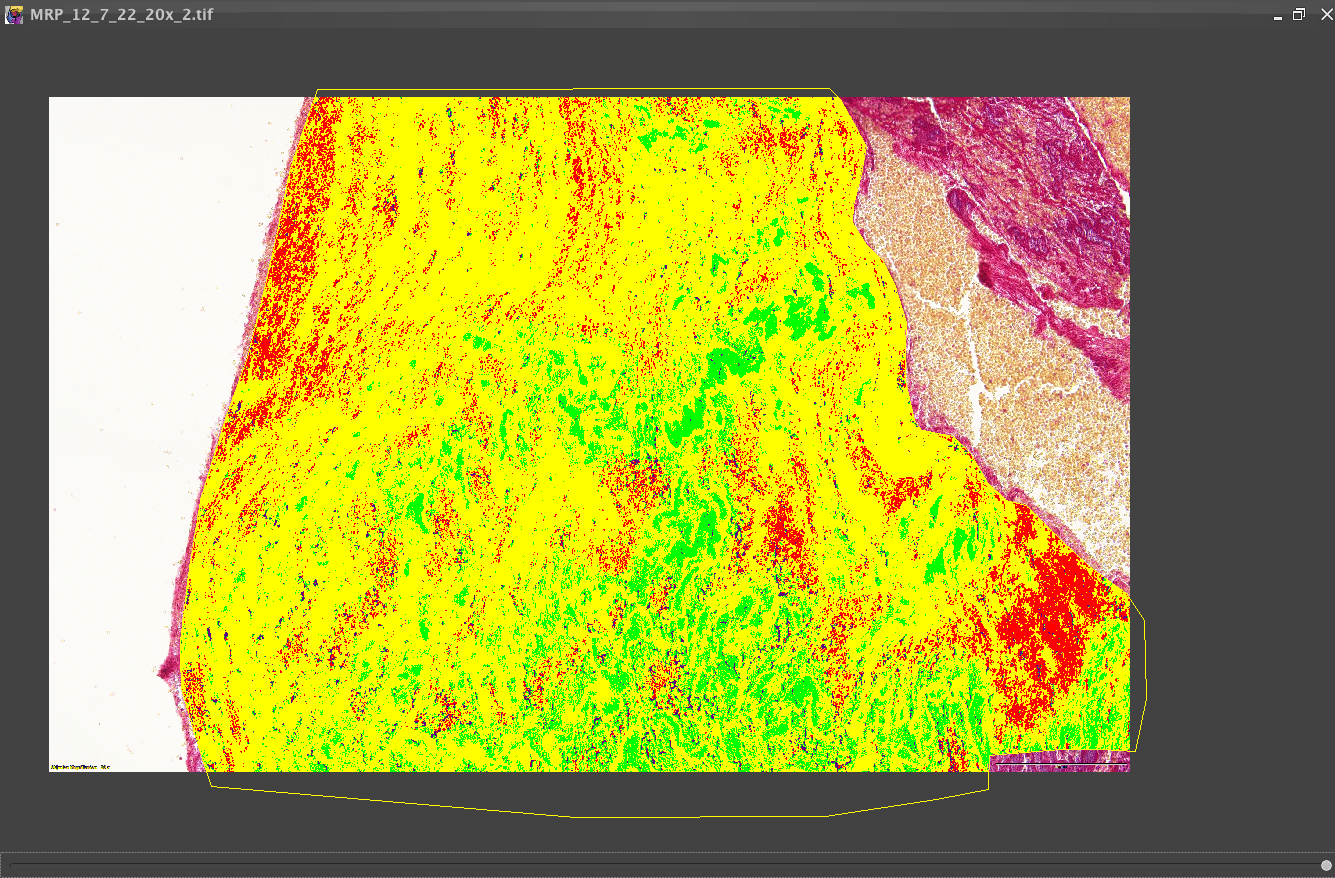
**

**Figure 17:** Spatial mapping of the clot constituents within the defined ROI.
